# Peak Expiratory Flow and Its Trajectories Associated With Frailty in Older Adults: A Prospective Cohort Study

**DOI:** 10.1002/jcsm.70123

**Published:** 2025-11-20

**Authors:** Dingchun Hou, Bo Liang, Kai Zhao, Ye Zhang, Minglang Lang, Zhiqin Xu, Hezhang Yun, Weijun Yang, Longqi Yu, Chang Liu

**Affiliations:** ^1^ Beijing Sport University Beijing China; ^2^ Institute of Population Research Peking University Beijing China; ^3^ School of Physical Education Zhejiang Guangsha Vocational and Technical University of Construction Jinhua China

**Keywords:** frailty, peak expiratory flow, prospective cohort, trajectory

## Abstract

**Background:**

Current evidence indicates an association between peak expiratory flow (PEF) and frailty in older adults, yet critical gaps persist regarding: the longitudinal dynamics of PEF trajectories, their temporal relationships with frailty progression and underlying biological mechanisms. This study aimed to investigate the associations of baseline PEF and its longitudinal trajectories with incident frailty risk, while elucidating potential mechanistic pathways in older adults.

**Methods:**

A prospective cohort study was conducted of general community‐dwelling adults aged ≥ 65 years from 2006 to 2022 in the Health and Retirement Study from the United States. PEF trajectories were identified using group‐based trajectory modelling. Frailty was assessed by the Fried frailty phenotype, encompassing five key components: weight loss, exhaustion, physical activity, gait speed and grip strength. Participants were classified as frailty if they met three or more. Cox proportional hazard models were applied to estimate the associations.

**Results:**

A total of 5686 participants with a median age of 72.5 (interquartile range: 68–77) years and 3262 (57.37%) females were included and followed up for 34 052 person‐years with an incidence density of frailty of 31.69 per 1000 person‐years. The proportion of White, Black and other ethnic populations was 83.45%, 12.56% and 3.99%, respectively. Compared with participants in the Q_5_ level of PEF at baseline, its Q_4_, Q_3_, Q_2_ and Q_1_ levels increased the risk of frailty by 38% (hazard ratio [HR] = 1.38, 95% confidence interval [CI]: 1.09–1.74), 86% (HR = 1.86, 95% CI: 1.46–2.38), 112% (HR = 2.12, 95% CI: 1.46–2.38) and 172% (HR = 2.72, 95% CI: 2.11–3.51), respectively. Similar associations were observed when using standardized residuals of PEF. A total of 1826 participants were included to model PEF trajectories over an 8‐year period, identifying five trajectories: high level (6.63%), upper‐moderate level (14.84%), moderate level (24.15%), lower‐moderate level (41.29%) and low level (13.09%). Compared with the high level, the low level increased the risk of frailty by 522% (HR = 6.22, 95% CI: 2.53–15.26). Low PEF over both short and extended periods was associated with an increased risk of various degrees of frailty phenotypes.

**Conclusions:**

Low PEF over both short and extended periods was significantly associated with an increased risk of frailty in older adults. These findings highlight the importance of monitoring pulmonary function as a potential marker for frailty risk and suggest that PEF may influence different frailty phenotypes to varying extents.

**Abbreviations**
ADL: activities of daily living
BMI: body mass index
CESD‐8: 8‐item version of the Center for Epidemiologic Studies Depression Scale
CI: confidence interval
CRP: C‐reactive protein
FFP: Fried frailty phenotype
GBTM: group‐based trajectory modelling
HbA1c: glycosylated haemoglobin A1c
HDL‐C: high‐density lipoprotein cholesterol
HR: hazard ratio
HRS: Health and Retirement Study
IQR: interquartile range
PEF: peak expiratory flow
RCS: restricted cubic spline
SR: standardized residual
TC: total cholesterol

## 1 Introduction

Frailty is a pathological manifestation of ageing, characterized by reduced physiological reserve across multiple organ systems, increased vulnerability to adverse health outcomes and impaired resilience to external stressors [1, 2]. Declines in lung function have been linked to an increased risk of respiratory, cardiovascular and metabolic dysfunctions, as well as heightened comorbidity and mortality rates [3]. A growing body of evidence supports an association between impaired lung function and the development of frailty [4, 5, 6, 7]. Consequently, age‐related lung function decline may serve as a critical marker for the early identification and diagnosis of frailty. Despite being the gold standard for lung function assessment, the necessity for specialized equipment and technical expertise with spirometry constrains its implementation in large‐scale epidemiological studies [8]. In contrast, peak expiratory flow (PEF) serves as a practical, cost‐effective and widely accessible metric for evaluating airway patency, respiratory muscle strength and overall pulmonary health [4, 9, 10]. Several studies indicated that lower PEF was associated with an increased risk of frailty [4, 6, 7, 11]. Furthermore, evidence indicated that PEF naturally declines with age and varies among individuals because of factors like sex and height [12]. Thus, the standardized residual (SR) percentiles and percent predicted of PEF have been proposed to further accurately reflect lung function in older adults [6, 12]. A prospective cohort study showed that PEF SR was considered a superior indicator in older Chinese [6]. However, significant racial differences in PEF highlight the necessity to evaluate the association between PEF SR and frailty risk among other races.

Divergent lung function trajectories are associated with varied health outcomes throughout the lifespan, highlighting the importance of monitoring these trajectories during early life stages [13, 14]. Lung function follows a three‐phase trajectory throughout the lifespan, with the decline phase typically commencing during middle adulthood [13, 14]. Although age‐related declines in lung function are associated with sarcopenia (a key component of frailty) across all age groups, this relationship with muscle mass is significantly stronger in older adults than in middle‐aged individuals [15]. Furthermore, pulmonary functional alterations in the elderly present greater pathophysiological complexity [16]. There has been a growing interest in monitoring and identifying lung function trajectories, which may provide valuable insights to enhance the understanding of the relationship between lung function and frailty [5, 14]. Although a prior study associated persistently low forced expiratory volume in 1 s with frailty, the absence of a prospective study design precludes causal inference, as the risk of reverse causality limits the accurate estimation of how lung function trajectories influence frailty risk [16]. Collectively, the surveillance and characterization of PEF trajectories are of great significance to detect early PEF changes and elucidate their implications for frailty development and progression. Conducting a prospective cohort study can provide valuable insights into the association between lung function trajectories and the risk of frailty in older adults.

Prior research revealed several potential mechanisms between PEF and frailty, including sarcopenia, inflammation, pulmonary infections due to impaired cough reflex and cognitive impairment [4, 6, 7]. Frailty is also considered the loss of resilience, and some phenotypes appear pivotal for the loss, the most important of which may be the deterioration of physical function, specifically decreased performance in measures such as skeletal muscle strength and mobility [1, 2]. Investigating how PEF measured over short and extended terms associates with specific frailty phenotypes can elucidate domain‐specific mechanisms and guide the development of phenotype‐targeted interventions.

In this study, we hypothesized that low PEF, PEF SR and poor PEF trajectories over an 8‐year period are associated with an increased risk of frailty and different frailty phenotypes among older adults. In order to test this hypothesis and provide a reference for the study of pathophysiological mechanisms, early prevention and clinical management of frailty, prospective cohorts were constructed using a nationwide population‐based longitudinal survey database from the United States.

## 2 Methods

### 2.1 Study Population

Prospective cohorts were conducted in this study. The data were from the Health and Retirement Study (HRS) from the United States, and detailed information on the study design for this longitudinal survey focusing on community‐dwelling adults aged 50 years or older has been published previously [17]. In brief, the HRS, a nationwide, prospective, population‐based longitudinal study, was approved by the Ethics Review Committees of the University of Michigan. Informed consent was obtained from each participant in the survey.

In the HRS, Wave 10 (2010) to Wave 13 (2016) were used as the baseline, with follow‐up surveys continuing until Wave 16 (2022). Wave 15 (2020) was not included in our study because of lacking physical measurement data. Participants were recruited if they were aged 65 years or older and had undergone physical examinations at baseline. Individuals with missing baseline PEF measurements or incomplete Fried frailty phenotype data (> 1 missing item) at baseline or during follow‐up were excluded. Frail individuals at baseline were excluded (*n* = 1454). Finally, 5686 participants from the HRS were included in the cohort to estimate the association of PEF at baseline with frailty risk. Figure S1 shows the flowchart depicting the sample selection process. According to the inclusion and exclusion criteria, there were no eligible participants included in Wave 13.

To estimate the association between PEF trajectories and the risk of frailty, we measured PEF from Wave 8 (2006) to Wave 13. Given the quadrennial assessment of the same cohort in the HRS, we included participants with available data of PEF at Wave 8, Wave 10 and Wave 12 for the trajectory analysis. Those with complete data at Wave 9 (2008), Wave 11 and Wave 13 were also included. Wave 12 and Wave 13 were used as the baseline, with follow‐ups continuing until Wave 16. Participants with complete data for three measurements of PEF were included at baseline. Other inclusion and exclusion criteria remained as stated above. However, according to the inclusion and exclusion criteria, there were no eligible participants included in Wave 13. As shown in Figure S2, 1826 participants were used to model PEF trajectories over an 8‐year period and included in the cohort.

### 2.2 Assessment of Frailty

The Fried frailty phenotype (FFP) was used to measure frailty by assessing features of slowness, weakness, exhaustion, inactivity and shrinking, which is currently the recommended international standard for frailty identification and assessment [18]. Each FFP item was assigned a score of 1 (presence) or 0 (absence), with the total score used to categorize participants into robust (score = 0), pre‐frailty (score = 1–2) and frailty (score = 3–5) [18]. The detailed information of the criteria for each item was shown in Table S1. Missing data rates for each item of the Fried frailty phenotype were low overall: weight loss (5.98%), exhaustion (0.20%), slowness (5.24%), weakness (2.16%) and inactivity (0%). Given the low proportion of missing data for each FFP item, random forest imputation using the missRanger package in R software was employed to maximize the sample size [19].

### 2.3 Assessment of PEF

PEF was assessed utilizing a Mini‐Wright peak flow metre (Clement Clarke International Ltd., Harlow, UK) coupled with a disposable mouthpiece, under the guidance of a trained interviewer. Three measurements were conducted with a 30‐s interval between each, and the maximum value was adopted for the respondent's PEF in our analyses. Two methodologies were applied to process the PEF data. First, participants were categorized into five groups based on the quintiles of measured PEF at baseline. Second, given prior research and acknowledging the physiological decrease in PEF associated with ageing, as well as its variability across individuals due to factors such as age, sex, stature, race and ethnicity, we operationalized the PEF variable through a two‐step methodology. Initially, we established a baseline healthy subsample comprising individuals who had never smoked and were free from any chronic lung diseases, asthma, heart conditions, stroke or cancer. Predicted PEF values were derived from sex‐specific predictive equations that incorporated age, stature, race and ethnicity, as determined within this healthy subsample [12, 20]. Subsequently, we calculated the SR percentiles of PEF from the normalization of the ratio (measured PEF minus predicted PEF) divided by the standard deviation of the residuals, with SR = 0 corresponding to the 50th percentile [12]. Taking into account the standard cut‐offs currently utilized in clinical practice and in previous studies, we divided the PEF SR percentiles into five categories (< 10th, ≥ 10th & < 30th, ≥ 30th & < 50th, ≥ 50th & < 80th and ≥ 80th percentiles) [4, 6].

### 2.4 Assessment of Covariates

The covariates included age, sex, race, place of residence, educational level, marital status, per capita household income, smoking status, alcohol consumption, body mass index, disability, number of chronic diseases, chronic respiratory disease status, depression, cognitive function, pain, systolic blood pressure, diastolic blood pressure, high‐density lipoprotein cholesterol (HDL‐C), total cholesterol (TC), C‐reactive protein (CRP) and glycosylated haemoglobin A1c (HbA1c). Measurements and cutoffs for covariates are presented in Table S1. Data for all covariates were collected at baseline. Given the low proportion of missing data for covariates, random forest imputation using the missRanger package in R software was applied to maximize the sample size [19].

### 2.5 Statistical Analysis

For descriptive analysis, continuous variables that were not normally distributed were presented as median and interquartile range (IQR). Categorical variables were presented as frequency and percentage. Univariate analysis was performed using the log‐rank test.

For the statistical inference, we used the Cox proportional hazard regression model to estimate the associations of PEF and PEF SR at baseline as an early exposure with subsequent frailty as the outcome. Schoenfeld residuals were used to test the proportional hazard assumption. Given the potential differences or interaction, subgroup analyses stratified by age and sex were further conducted. To explore potential mechanisms between PEF and frailty, robust individuals were included at baseline to construct cohorts to assess the associations of PEF and PEF SR at baseline with specific frailty phenotypes. Restricted cubic splines (RCS) with five knots were employed to explore the dose–response relationship between PEF and the risk of frailty. Given the significant sex differences in PEF distribution, RCS with five knots were applied in the sex‐stratified analysis. All models referenced the Q_5_ level of PEF or 80th percentile of PEF SR and more, adjusted for baseline covariates that were significantly associated with frailty in univariate analyses (*p* < 0.05) and presented results as hazard ratios (HRs) with 95% confidence intervals (95% CI).

PEF trajectories were modelled with group‐based trajectory modelling (GBTM) by the Traj package of STATA. GBTM is a semi‐parametric procedure designed to identify subgroups with similar changes over time or age [21]. The detailed procedure has been described previously [21]. All possible models with a polynomial order of 1 to 3 and the number of trajectory groups of 2 to 5 were built. We selected the model with the bayesian information criterion (BIC) closer to zero and sufficient sample size (at least 5% of the total) in each trajectory group. Model adequacy is indicated by (1) an average group posterior probability of greater than 0.7 for each group, (2) an odd of correct classification of 5 or more for all groups, (3) an entropy value above 0.8 and (4) a close correspondence between the estimated group probabilities and the proportion of group assignment using the maximum posterior probability assignment rule [21]. Cox proportional hazard regression model was used to estimate the association between PEF trajectories and subsequent frailty. Furthermore, robust individuals were included at baseline to model PEF trajectories and construct cohorts to assess the association between PEF trajectories and specific frailty phenotypes.

To ascertain the robustness of our findings, we conducted several sensitivity analyses: (1) Two cohorts, with frailty as the outcome, were constructed by separately including individuals who were robust or pre‐frail at baseline, to estimate the association of baseline PEF and PEF SR with frailty. (2) Given that smoking is a strong confounder in the studied association, we have repeated the analyses including only individuals who have never smoked, (3) We excluded participants with respiratory diseases at baseline and repeated the analyses. (4) Considering the potential effect of the imputation for missing values of FFP, we repeated the analyses among participants without missing values of FFP.

All analyses were performed using STATA, version 17.0 and R software, version 4.4.1. *p* < 0.05 (two‐tailed) was considered statistically significant.

## 3 Results

### 3.1 Baseline and Follow‐Up Characteristics of Study Populations

A total of 5686 eligible participants with a median age of 72.5 (IQR: 68–77) and 3262 (57.37%) females were included in our study and followed up for 34 052 person‐years, with an incidence density of frailty at 31.69 per 1000 person‐years. The proportion of White, Black and other ethnic populations was 83.45%, 12.56% and 3.99%, respectively. The baseline characteristics of the study population with and without frailty during follow‐up are detailed in Table 1.

**TABLE 1:** Distribution characteristics of participants with and without frailty during follow‐up (*n* = 5686).

Characteristics	Nonfrailty (*n* = 4607) *n* (%)	Frailty (*n* = 1079) *n* (%)	*p*
Age			< 0.001
≥ 65 & < 75 years	2961 (85.16)	516 (14.84)	
≥ 75 years	1646 (74.51)	563 (25.49)	
Sex			0.003
Male	2009 (82.88)	415 (17.12)	
Female	2598 (79.64)	664 (20.36)	
Race			< 0.001
White	3897 (82.13)	848 (17.87)	
Black	532 (74.51)	182 (25.49)	
Others	178 (78.41)	49 (21.59)	
Place of residence			0.794
Urban	2202 (81.02)	516 (18.98)	
Suburban	1091 (80.40)	266 (19.60)	
Exurban	1314 (81.56)	297 (18.44)	
Educational level			< 0.001
High school or below	2255 (76.70)	685 (23.30)	
Above high school	2352 (85.65)	394 (14.35)	
Marital status			< 0.001
Married or partnered	3130 (82.85)	648 (17.15)	
Others	1477 (77.41)	431 (22.59)	
Per capita household income ($)			< 0.001
Q_1_	1017 (71.57)	404 (28.43)	
Q_2_	1139 (80.15)	282 (19.85)	
Q_3_	1207 (84.94)	214 (15.06)	
Q_4_	1244 (87.42)	179 (12.58)	
Smoking status			0.035
Never	2111 (81.01)	495 (18.99)	
Former	2122 (81.65)	477 (18.35)	
Current	374 (77.75)	107 (22.25)	
Alcohol consumption			< 0.001
No	1921 (77.24)	566 (22.76)	
Yes	2686 (83.96)	513 (16.04)	
Body mass index (kg/m^2^)			< 0.001
< 25	1059 (86.38)	167 (13.62)	
≥ 25 & < 30	1751 (83.06)	357 (16.94)	
≥ 30	1797 (76.40)	555 (23.60)	
Disability			< 0.001
No	4236 (83.03)	866 (16.97)	
Yes	371 (63.53)	213 (36.47)	
Number of chronic diseases			< 0.001
0	477 (91.20)	46 (8.80)	
1	1160 (88.35)	153 (11.65)	
≥ 2	2970 (77.14)	880 (22.86)	
Chronic respiratory disease status			< 0.001
No chronic respiratory diseases	4244 (81.91)	937 (18.09)	
Chronic respiratory diseases with treatment	212 (69.74)	92 (30.26)	
Chronic respiratory diseases without treatment	151 (75.12)	50 (24.88)	
Depression			< 0.001
No	4293 (82.27)	925 (17.73)	
Yes	314 (67.09)	154 (32.91)	
Cognitive function			< 0.001
Normal	3948 (83.22)	796 (16.78)	
Mild cognitive impairment	581 (70.25)	246 (29.75)	
Dementia	78 (67.83)	37 (32.17)	
Pain			< 0.001
No	3233 (84.48)	594 (15.52)	
Yes	1374 (73.91)	485 (26.09)	
Systolic blood pressure (mmHg)			0.144
< 140	3184 (81.49)	723 (18.51)	
≥ 140	1423 (79.99)	356 (20.01)	
Diastolic blood pressure (mmHg)			0.174
< 90	4017 (80.91)	948 (19.09)	
≥ 90	590 (81.83)	131 (18.17)	
High‐density lipoprotein cholesterol (mg/L)			0.109
< 50 for males/< 40 for females	3294 (81.47)	749 (18.53)	
≥ 50 for males/≥ 40 for females	1313 (79.91)	330 (20.09)	
Total cholesterol (mg/L)			0.003
< 200	2664 (79.86)	672 (20.14)	
≥ 200	1943 (82.68)	407 (17.32)	
C‐reactive protein (mg/L)			< 0.001
3	3102 (82.74)	647 (17.26)	
≥ 3	1505 (77.70)	432 (22.30)	
Glycosylated haemoglobin A1c (%)			< 0.001
< 6.5	3996 (82.72)	835 (17.28)	
≥ 6.5	611 (71.46)	244 (28.54)	
Apolipoprotein E gene			0.235
Non‐ε4 carriers	3481 (81.01)	816 (18.99)	
Heterozygous ɛ4 carriers	1045 (81.45)	238 (18.55)	
Homozygous ɛ4 carriers	81 (76.42)	25 (23.58)	
Peak expiratory flow			< 0.001
Q_1_	744 (70.32)	314 (29.68)	
Q_2_	855 (78.30)	237 (21.70)	
Q_3_	947 (81.15)	220 (18.85)	
Q_4_	1027 (84.81)	184 (15.19)	
Q_5_	1034 (89.29)	124 (10.71)	
Peak expiratory flow standard residual percentiles			< 0.001
≥ 80th	977 (85.85)	161 (14.15)	
≥ 50th & < 80th	1415 (82.99)	290 (17.01)	
≥ 30th & < 50th	931 (81.81)	207 (18.19)	
≥ 10th & < 30th	879 (77.31)	258 (22.69)	
< 10th	405 (71.30)	163 (28.70)	

### 3.2 Trajectories of PEF

**FIGURE 1:**
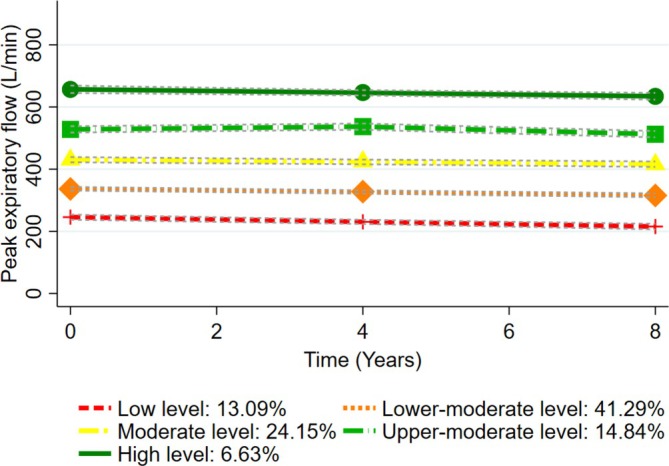
Trajectories of peak expiratory flow over an 8‐year period among older adults (*n* = 1826).

A total of 1826 participants with a median age of 74 (IQR: 69–79) years and 1077 (58.98%) females were used to model trajectories of measured PEF over an 8‐year period. Table S2 shows the diagnostics of all possible models. Baseline characteristics of the participants included in the trajectory analysis of PEF are shown in Table S3. As shown in Figure 1, five PEF trajectories were identified: high level (6.63%), upper‐moderate level (14.84%), moderate level (24.15%), lower‐moderate level (41.29%) and low level (13.09%).

### 3.3 Associations of PEF at Baseline and Trajectories With Frailty

**FIGURE 2:**
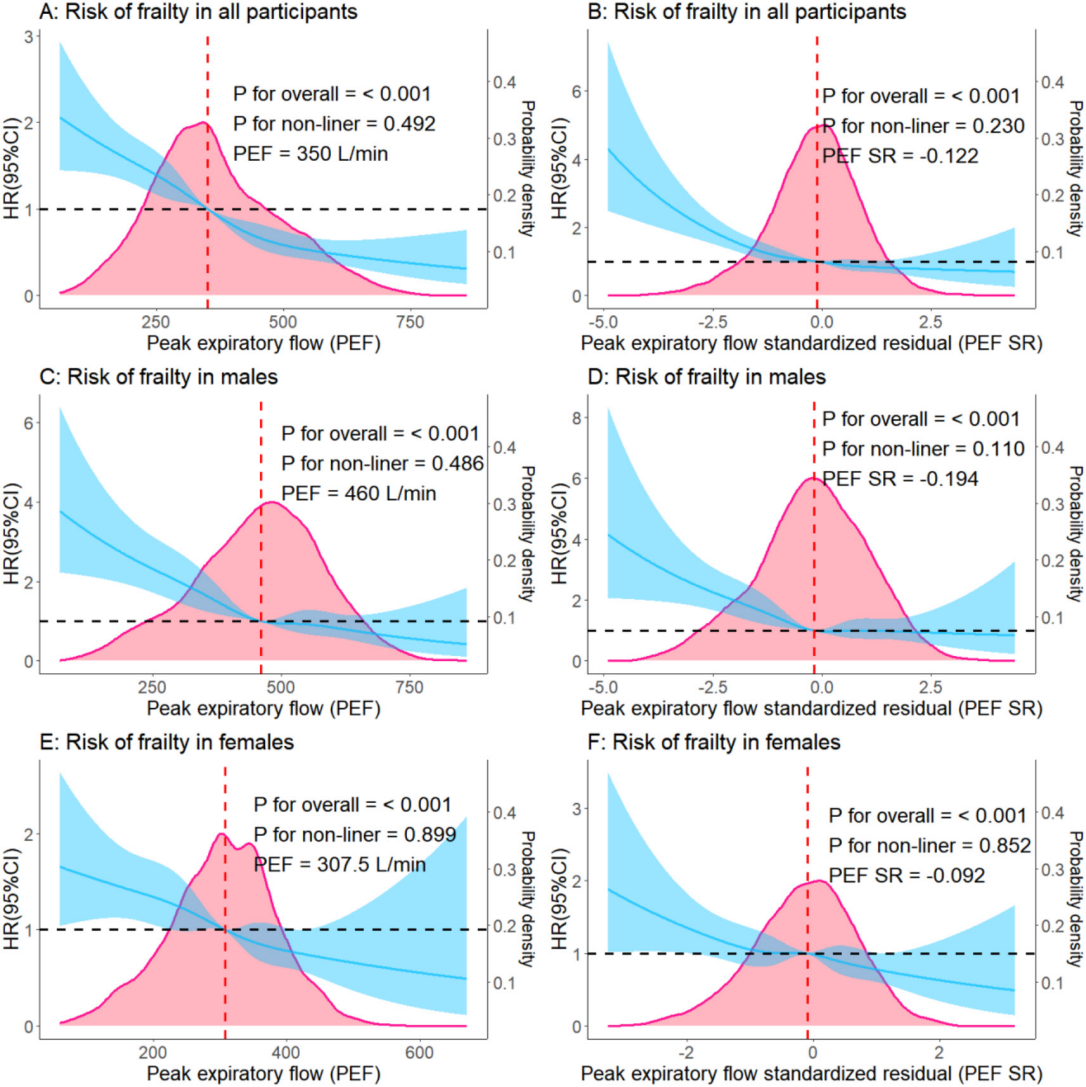
The association between peak expiratory flow and frailty among older adults. *Note:* CI: confidence interval; HR: hazard ratio. For the analysis in all participants, hazard ratios were adjusted for age, sex, race, educational level, marital status, per capita household income, smoking status, alcohol consumption, body mass index, disability, number of chronic diseases, chronic respiratory disease status, depression, cognitive function, pain, total cholesterol, C‐reactive protein and glycosylated haemoglobin A1c. For the sex‐stratified analysis, hazard ratios were adjusted for covariates mentioned above, except for sex.

Table 2 shows the associations of PEF and PEF SR at baseline with frailty risk. Compared with the Q_5_ level of PEF, its Q_4_, Q_3_, Q_2_ and Q_1_ levels increased the risk of frailty by 38% (HR = 1.38, 95% CI: 1.09–1.74), 86% (HR = 1.86, 95% CI: 1.46–2.38), 112% (HR = 2.12, 95% CI: 1.46–2.38) and 172% (HR = 2.72, 95% CI: 2.11–3.51), respectively. Compared with the highest level of PEF SR (≥ 80th percentile), lower PEF SR levels (≥ 10th & < 30th, and < 10th percentile) increased the risk of frailty by 43% (HR = 1.43, 95% CI: 1.17–1.75) and 107% (HR = 2.07, 95% CI: 1.64–2.60), respectively. As shown in Figure S3, no significant interaction between age or sex and PEF or PEF SR on the frailty risk were observed (*P*_for interaction_ ≥ 0.05). Figure 2 shows a dose–response relationship between measured PEF or PEF SR at baseline and the risk of frailty revealed by RCS curves. Furthermore, significant sex‐specific differences were observed in the distribution curves of PEF. The optimal bivariate cut‐off value for frailty risk, at which the HR was equal to 1, was 460 L/min for men and 307.5 L/min for women. Although the association between PEF and the risk of frailty remained largely consistent across both sexes, and a significant linear relationship was observed in both men and women (*P*_for non‐linear_ > 0.05).

**TABLE 2:** Association of peak expiratory flow and its trajectories with frailty risk in the Cox proportional hazard regression model.

Peak expiratory flow	Person‐years of follow‐up	Incidence density (per 1000 person‐years)	HR (95% CI)
Measured values at baseline (*n* = 5686)			
Q_1_	5644	55.63	**2.72 (2.11–3.51)**
Q_2_	6372	37.19	**2.12 (1.64–2.73)**
Q_3_	7180	30.64	**1.86 (1.46–2.38)**
Q_4_	7392	24.89	**1.38 (1.09–1.74)**
Q_5_	7464	16.61	Reference
Standard residual percentiles at baseline (*n* = 5686)			
≥ 80th	7076	22.75	Reference
≥ 50th & < 80th	10 408	27.86	1.18 (0.97–1.44)
≥ 30th & < 50th	6956	29.76	1.22 (0.99–1.50)
≥ 10th & < 30th	6596	39.11	**1.43 (1.17–1.75)**
< 10th	3016	54.05	**2.07 (1.64–2.60)**
Peak expiratory flow trajectories (*n* = 1826)			
High	2306	11.27	Reference
Upper‐moderate	5440	16.54	2.31 (0.96–5.55)
Moderate	8856	25.41	**3.28 (1.40–7.65)**
Lower‐moderate	12 156	33.48	**5.14 (2.18–12.15)**
Low	3962	56.54	**6.22 (2.53–15.26)**

*Note:* Boldface: *p* < 0.05. For the analyses of peak expiratory flow at baseline, hazard ratios were adjusted for age, sex, race, educational level, marital status, per capita household income, smoking status, alcohol consumption, body mass index, disability, number of chronic diseases, chronic respiratory disease status, depression, cognitive function, pain, total cholesterol, C‐reactive protein and glycosylated haemoglobin A1c. For the analyses of peak expiratory flow trajectories, hazard ratios were adjusted for age, sex, race, educational level, marital status, per capita household income, alcohol consumption, body mass index, disability, number of chronic diseases, chronic respiratory disease status, depression, cognitive function, pain, total cholesterol, C‐reactive protein and glycosylated haemoglobin A1c.

Abbreviations: CI: confidence interval; HR: hazard ratio.

Compared with the high level PEF trajectory, moderate level, lower‐moderate level and low level trajectories increased the risk of frailty by 228% (HR = 3.28, 95% CI: 1.40–7.65), 414% (HR = 5.14, 95% CI: 2.18–12.15) and 522% (HR = 6.22, 95% CI: 2.53–15.26).

### 3.4 Associations of PEF at Baseline and Trajectories With Specific Frailty Phenotypes

Table 3 shows the associations of PEF and PEF SR at baseline, along with PEF trajectories, with specific frailty phenotypes. Among the five dimensions of frailty, a lower level of PEF or PEF SR at baseline was associated with slowness, weakness and exhaustion (*p* < 0.05). Furthermore, PEF trajectories over an 8‐year period were associated with these dimensions (*p* < 0.05). Although PEF at baseline and PEF trajectories were not associated with inactivity (*p* > 0.05), lower levels of PEF SR at baseline were associated with an increased risk of inactivity (*p* < 0.05). PEF and PEF SR at baseline, as well as PEF trajectories, were not associated with shrinking (*p* > 0.05).

**TABLE 3:** Association of peak expiratory flow and its trajectories with items of the Fried frailty phenotype in the Cox proportional hazard regression model, HR (95% CI).

Peak expiratory flow	Fried frailty phenotype				
	Slowness^a^	Weakness^b^	Exhaustion^c^	Inactivity^d^	Shrinking^e^
Measured values at baseline (*n* = 2712)					
Q_1_	**1.90 (1.44–2.50)**	**1.97 (1.55–2.51)**	**1.71 (1.25–2.34)**	1.28 (0.93–1.76)	1.72 (0.61–4.85)
Q_2_	**1.42 (1.08–1.86)**	**1.54 (1.22–1.94)**	**1.44 (1.07–1.95)**	0.99 (0.73–1.36)	0.50 (0.14–1.80)
Q_3_	**1.50 (1.17–1.92)**	**1.40 (1.11–1.77)**	**1.40 (1.03–1.89)**	0.96 (0.70–1.31)	0.56 (0.19–1.62)
Q_4_	1.05 (0.83–1.34)	**1.29 (1.01–1.65)**	**1.48 (1.10–2.01)**	0.91 (0.66–1.25)	1.07 (0.48–2.37)
Q_5_	Reference	Reference	Reference	Reference	Reference
Standard residual percentiles at baseline (*n* = 2712)					
≥ 80th	Reference	Reference	Reference	Reference	Reference
≥ 50th & < 80th	1.21 (0.99–1.49)	**1.26 (1.01–1.56)**	1.12 (0.85–1.48)	1.04 (0.77–1.39)	0.44 (0.16–1.21)
≥ 30th & < 50th	1.18 (0.95–1.48)	**1.29 (1.02–1.64)**	1.25 (0.93–1.67)	1.09 (0.79–1.49)	1.04 (0.43–2.51)
≥ 10th & < 30th	**1.40 (1.13–1.75)**	**1.62 (1.29–2.04)**	**1.39 (1.05–1.86)**	1.27 (0.93–1.72)	1.46 (0.64–3.33)
< 10th	**2.03 (1.58–2.61)**	**1.77 (1.36–2.30)**	**1.60 (1.14–2.25)**	**1.76 (1.25–2.49)**	1.50 (0.57–3.92)
Peak expiratory flow trajectories (*n* = 889)					
High	Reference	Reference	Reference	Reference	Reference
Upper‐moderate	1.22 (0.70–2.12)	2.35 (1.17–4.73)	**2.24 (1.02–4.92)**	1.37 (0.63–3.00)	2.87 (0.32–25.89)
Moderate	1.62 (0.95–2.78)	**2.99 (1.49–6.03)**	2.08 (0.92–4.75)	1.20 (0.57–2.56)	5.58 (0.61–51.17)
Lower‐moderate	**2.31 (1.29–4.14)**	**4.40 (2.11–9.18)**	1.69 (0.71–4.04)	1.46 (0.71–3.00)	1.43 (0.09–22.52)
Low	**2.79 (1.40–5.56)**	**6.38 (2.76–14.73)**	**2.75 (1.01–7.50)**	1.55 (0.64–3.75)	3.40 (0.11–105.62)

*Note:* Boldface: *p* < 0.05.

Abbreviations: CI: confidence interval; HR: hazard ratio.

^a^ For analysis of peak expiratory flow at baseline, hazard ratios were adjusted for age, sex, race, educational level, marital status, per capita household income, alcohol consumption, body mass index, disability, number of chronic diseases, chronic respiratory disease status, cognitive function, systolic blood pressure, C‐reactive protein and glycosylated haemoglobin A1c; for analysis of peak expiratory flow trajectories, hazard ratios were adjusted for age, sex, race, educational level, marital status, per capita household income, alcohol consumption, body mass index, disability, number of chronic diseases, pain and glycosylated haemoglobin A1c.

^b^ For analysis of peak expiratory flow at baseline, hazard ratios were adjusted for age, educational level, marital status, per capita household income, body mass index, number of chronic diseases, pain, cognitive function and glycosylated haemoglobin A1c; for analysis of peak expiratory flow trajectories, hazard ratios were adjusted for age, sex, educational level, marital status, per capita household income, body mass index, number of chronic diseases, chronic respiratory disease status and C‐reactive protein.

^c^ For analysis of peak expiratory flow at baseline, hazard ratios were adjusted for age, race, educational level, marital status, per capita household income, smoking status, disability, number of chronic diseases, chronic respiratory disease status, depression, cognitive function, pain, systolic blood pressure, C‐reactive protein and glycosylated haemoglobin A1c. For analysis of peak expiratory flow trajectories, hazard ratios were adjusted for sex, race, marital status, disability, number of chronic diseases, chronic respiratory disease status, depression and pain.

^d^ For analysis of peak expiratory flow at baseline, hazard ratios were adjusted for age, race, educational level, marital status, per capita household income, smoking status, alcohol consumption, body mass index, number of chronic diseases, chronic respiratory disease status, cognitive function, systolic blood pressure, C‐reactive protein and glycosylated haemoglobin A1c. For analysis of peak expiratory flow trajectories, hazard ratios were adjusted for age, race, educational level, marital status, per capita household income, alcohol consumption, body mass index, number of chronic diseases, cognitive function, pain, C‐reactive protein and glycosylated haemoglobin A1c.

^e^ For analysis of peak expiratory flow at baseline, hazard ratios were adjusted for sex, educational level, body mass index and diastolic blood pressure; for analysis of peak expiratory flow trajectories, hazard ratios were adjusted for sex, place of residence, disability, chronic respiratory disease status, C‐reactive protein and glycosylated haemoglobin A1c.

### 3.5 Sensitivity Analyses

Regardless of whether cohorts were constructed using robust or pre‐frail individuals at baseline, the findings remained consistent with the main analysis results (Table S4). When we repeated the analyses among individuals who never smoked, the findings were consistent with the main analysis (Table S7). When we repeated the analyses among individuals without respiratory diseases at baseline, the results were consistent with the main analysis (Table S9). Furthermore, when we repeated the analyses among participants without missing values of FFP, the results were in line with the main analysis (Table S11). Overall, the sensitivity analyses substantiated the robustness of the main analysis results.

## 4 Discussion

To our best knowledge, this is a large‐scale prospective cohort study for the association of PEF and its trajectories with the risk of frailty and different frailty phenotypes. Our findings showed that a higher level of PEF at baseline was associated with a reduced risk of frailty among older adults, with a dose–response relationship. Five PEF trajectories were modelled over an 8‐year period, indicating a pattern of gradual decline followed by stabilization in PEF progression with age. Sustained long‐term stability in lower PEF levels was associated with an increased risk of frailty. PEF at baseline and its trajectories demonstrated distinct associations with different frailty phenotypes.

Lung function is not merely a predictor of pulmonary diseases but also associated with multisystem function [13]. Evidence demonstrated that lung function was associated with cognitive impairment, disability and mortality, suggesting its significant potential to assess broader health outcomes [10, 22, 23]. Compared with other lung function indicators, PEF does not fully reflect other aspects of lung function, such as small airway function and lung volume; however, its sensitivity to airway obstruction is higher, which can capture the early changes of airway function [3, 12]. Owing to its low cost and operational simplicity, PEF is well‐suited for a wide range of applications, including routine clinical assessment, daily home monitoring and large‐scale epidemiological research [12, 20]. Prior research has indicated the importance of monitoring lung function trajectories [14]. The normal lung function trajectory from birth to death has three phases: a growth phase (from birth to early adulthood), a plateau phase (that lasts for a few years) and a decline phase resulting from physiological lung aging [13]. Although the sensitivities of various lung function indicators may differ, a decline in lung function has been noted in several studies, often beginning as early as midlife [13, 22, 24]. However, more attention should be paid to delineating the PEF trajectories. A longitudinal study showed that all PEF trajectories exhibited similar rates of linear decline with age, differing primarily in their intercepts [24]. Similarly, our findings modelled five PEF trajectories, indicating a slow progression of PEF decline with age over an 8‐year period.

Accumulating evidence supports an association between reduced PEF and an increased risk of frailty [4, 6, 7, 11]. Although a prior study reported inconsistent findings, potentially due to its cross‐sectional design and specific setting [25], several cohort studies have corroborated our findings [4, 6]. PEF has been shown to exert a significant effect on frailty risk among middle‐aged and older Europeans, based on a recent Mendelian randomization study [7]. Although most studies have assessed PEF at baseline, limited evidence exists regarding the association between PEF progression across prolonged periods and frailty. Our study modelled 8‐year PEF trajectories and showed that PEF exhibited a gradual age‐related decline, characterized by poor reversibility. This suggested that long‐term monitoring of PEF may serve as a more robust predictor for frailty. Collectively, our study provides further insights into the evidence highlighting that low PEF over short and extended periods is associated with frailty among older adults. Notably, the trajectory of lung function decline should be interpreted as a potential biomarker extending beyond physiological ageing, reflecting concurrent systemic functional deterioration and pathological ageing.

Additionally, prior research demonstrated the sex‐specific differences in the distribution of PEF, indicating that PEF in men may be significantly higher than that in women [12, 24]. Although our results revealed that the association between PEF and the risk of frailty remained largely consistent across both sexes, the observed dissociation between significant sex differences in PEF distribution and the absence of sex‐specific associations between PEF and the risk of frailty may suggest distinct biological and pathological mechanisms underlying these phenomena. Despite physiological sex‐based variations in PEF, the uniform association between PEF and the risk of frailty across sexes implies that shared pathways, such as systemic inflammation, sarcopenia‐related muscle depletion or oxidative stress, may mediate this association independently of sex [26, 27]. This indicates that although sex‐specific reference values for PEF remain clinically relevant, its role as a frailty biomarker operates through mechanisms transcending sexual dimorphism. Our findings supported PEF's utility as a universal risk indicator, emphasizing the need to prioritize individual PEF trajectory monitoring over cross‐sex comparisons in frailty prediction. Future research should investigate sex‐neutral pathological cascades linking respiratory function decline to multisystem frailty manifestations.

The precise biological mechanisms underlying the association of PEF with frailty remain poorly understood. Our study investigated the associations between PEF and different frailty phenotypes to explore and examine potential mechanisms. With five phenotypes reflecting different physiological or clinical dimensions of frailty [1], our findings showed that low PEF over short or extended periods was associated with varying degrees of increased risk for slowness, weakness, exhaustion and inactivity. It is well established that slowness and weakness, assessed by gait speed and handgrip strength, respectively, are core components of sarcopenia, which is marked by progressive muscle loss and declining physical function [28]. On the one hand, as a key pathophysiological mechanism in frailty progression, sarcopenia can exacerbate exhaustion and reduce the body's sensitivity threshold to fatigue [29]. Furthermore, sarcopenia and exhaustion can limit the body's capacity for physical activity, affecting both its duration and intensity [30, 31]. The experience of exhaustion, particularly its physical manifestation as muscle fatigue, may represent a key mediating pathway through which sarcopenia leads to diminished physical activity [29]. On the other hand, both exhaustion and low physical activity may exacerbate sarcopenia, which shares multiple biomarkers with frailty [29, 30, 32]. Therefore, a potential vicious cycle could exist across different frailty phenotypes [1, 2], with PEF linked to multiple aspects within this cycle. However, our results showed that the association between PEF and shrinking did not reach statistical significance. Considering that PEF was associated with other frailty phenotypes, our study suggested that PEF may be more sensitive for detecting reductions in lean body mass, while being less sensitive to reductions in overall body weight. Therefore, low PEF may be associated with sarcopenic obesity, a pathological interaction between adipose tissue and skeletal muscle dysfunction, which is linked to the development of frailty [33, 34, 35]. As an indicator of diminished respiratory muscle strength, lower PEF has been shown to correlate more strongly with muscle mass than forced expiratory volume in 1 s [9]. The exertion of a PEF manoeuvre requires the coordinated action of primary (internal intercostals, intercostalis intimi, subcostals) and accessory (abdominal wall musculature) expiratory muscles. Therefore, PEF serves as a direct measure of this muscle function, explaining its association with total body muscle mass [36, 37]. Furthermore, the low incidence density of shrinking in this study could introduce potential biases into our estimation of the association.

Several physiological mechanisms underscore the critical role of PEF within the vicious cycle of frailty phenotypes that drive the cycle forward and progression. Low PEF clinically indicates airway obstruction and reduced respiratory muscle strength and mass [4, 9]. These age‐related alterations in respiratory dynamics, driven by chronic inflammation, mechanical restrictions, pulmonary infections and parenchymal lung damage, are directly or indirectly reflected by PEF [4, 38, 39]. As an indicator of respiratory system impairment, low PEF reflects a decline in the body's oxygen uptake capacity [9, 12]. Collectively, low PEF may suggest chronic inflammation, skeletal muscle dysfunction and energy metabolism abnormality. These pathophysiological changes provide a link between PEF and the development of frailty. The consistent findings in our trajectory analysis support these underlying mechanisms.

This study had several strengths. First, the construction of prospective cohorts with a relatively longer follow‐up period from a nationwide survey with large sample sizes mitigated the risk of reverse causality and bolstered the generalizability of our findings. Second, lung‐function trajectories are an important indicator for adverse health outcomes [14]. Our study observed the developmental trajectory of PEF over an 8‐year period and estimated their association with the risk of frailty. Third, we explored the association of PEF with different frailty phenotypes to examine the potential mechanisms between PEF and frailty. Our study provided further evidence to support the association between lung function and frailty and their underlying mechanisms. This study also had several limitations that warrant discussion. First, although prior research suggested that two spirometric measurements over 5 years may capture clinically relevant short‐term lung function trajectories in older adults [16], our 8‐year period with three observation time points might still inadequately characterize age‐related pulmonary decline in an extended term. Furthermore, evidence indicated that the lung function decline typically begins in midlife [13]. Future research could extend the observation period, particularly for monitoring lung function during the early stage of life. Second, selection bias was present because of sample selection and attrition over the follow‐up period. Although missing covariate values were handled through random forest imputation, the study remains subject to inherent limitations of longitudinal designs, including non‐random attrition from loss to follow‐up and mortality. Finally, there may be unmeasured confounding factors that were not adjusted for in our analyses, potentially introducing confounding bias. For instance, although studies have shown an association between the gene SMP30 and frailty [40], this gene was not included in our analyses because of the lack of data.

In summary, our study carries significant clinical and public health implications. First, our findings affirm the association of low PEF over short and extended periods with an increased risk of frailty. We also investigated the associations of PEF and its trajectories with specific frailty phenotypes, which enhanced risk stratification for frailty. PEF may serve as a simple, objective biomarker for identifying individuals at high risk for specific frailty phenotypes, including weakness, slowness, exhaustion and inactivity, enabling earlier and more precise risk assessment before full frailty onset and allowing for timely, lower‐cost preventative strategies. Additionally, the easy measurement of PEF provides a low‐cost, rapid and easily deployable tool for large‐scale community or primary care screening to identify pre‐frail individuals or those with specific high‐risk phenotypes, particularly in resource‐limited settings. Given the gradual decline and poor reversibility of lung function, monitoring and identifying long‐term PEF trajectories should be prioritized for frailty prevention efforts. Those who are robust or pre‐frail may also benefit from PEF evaluation to identify at‐risk individuals early. Collectively, our study provided potential evidence for integrating respiratory function assessment, such as PEF, into routine frailty screening and geriatric care, promoting holistic ‘muscle‐respiratory‐functional’ health management models. Pulmonary rehabilitation programs integrating inspiratory muscle training, aerobic conditioning and nutritional supplementation could be evaluated in robust and pre‐frail older adults exhibiting early PEF decline. Pharmacological strategies—such as trials of anti‐inflammatory agents in high‐inflammatory phenotypes or bronchodilators in rapid PEF decliners—represent another actionable avenue. Concurrently, community‐based lifestyle interventions targeting physical activity adherence and smoking cessation could be tested for their capacity to preserve PEF trajectories. Based on the phenotype‐specific associations observed in our study, future research could focus on the predictive value of respiratory sarcopenia for frailty risk. Furthermore, future research could conduct a series of intervention studies, such as sport and dance, to identify the most effective intervention measures for different phenotypes of frailty, thereby forming a multi‐dimensional and comprehensive clinical intervention plan for frailty.

## 5 Conclusions

In conclusion, low PEF at baseline was associated with an increased risk of frailty in older adults. With the gradual decline and stability with age, the trajectories of PEF were associated with frailty risk. Furthermore, PEF was associated to varying degrees with different frailty phenotypes. PEF and its trajectories stand out as promising predictors for early screening of frailty, assessment of prognosis and a potential therapeutic target for the development of intervention strategies.

## Ethics Statement

All human and animal studies have been approved by the appropriate ethics committee and have therefore been performed in accordance with the ethical standards laid down in the 1964 Declaration of Helsinki and its later amendments.

## Consent

Informed consent for publication was obtained from all participants.

## Conflicts of Interest

The authors declare no conflicts of interest.

## Funding

The work was supported by the Peking University Medical‐Qingyan Boshi Joint Laboratory for skin nutrition and anti‐aging (No. L202206)，the National Teams Science and Technology Support Program for Youth of the General Administration of Sport of China (No.24QN017), the Applied Research in the Intersection of Sport an Dance (No.BSU20240129) and the High‐Quality Development Cultivation Program of the Beijing Sport University (No.2024SKPY18), and in part by the Peking University Health Science Center for the Study on the effects of anti‐aging nutritional interventions on athletes' skin health (No. YFF24000662 and No. BSU20240500). The work was supported by the Beijing Higher Education Society’s Sub‐Committee on University Student Employment and Entrepreneurship Research under the project titled “Research on the Transformation Path and Mechanism of Innovation and Entrepreneurship Competition Achievements among University Students—Based on the Practice of Sports Universities” (Project No. DXSJCFHMS2025023).

## Supporting information


**Figure S1:** Selection flow of the study population for the association between peak expiratory flow at baseline and the risk of frailty in the HRS.
**Figure S2:** Selection flow of the study population for the association between peak expiratory flow trajectories and the risk of frailty in the HRS.
**Figure S3:** Subgroup analyses stratified by age and sex for the association between peak expiratory flow and the risk of frailty.
**Table S1:** The criteria and cutoffs for Fried frailty phenotype, peak expiratory flow and covariates.
**Table S2:** Diagnostics of models with all possible combinations of polynomial order and number of groups for the analysis of frailty.
**Table S3:** Distribution characteristics of peak expiratory flow trajectories among the American population at baseline (*n* = 1826).
**Table S4:** Association of peak expiratory flow at baseline with frailty in the Cox proportional hazard regression model for sensitivity analysis, HR (95% CI).
**Table S5:** Diagnostics of models with all possible combinations of polynomial order and number of groups for the analysis of different frailty phenotypes.
**Table S6:** Diagnostics of models with all possible combinations of polynomial order and number of groups for the analysis of frailty among never smokers.
**Table S7:** Association of peak expiratory flow at baseline and its trajectories with frailty risk among never smokers.
**Table S8:** Diagnostics of models with all possible combinations of polynomial order and number of groups for the analysis of frailty among participants without respiratory diseases at baseline.
**Table S9:** Association of peak expiratory flow at baseline and its trajectories with frailty risk among participants without respiratory diseases at baseline.
**Table S10:** Diagnostics of models with all possible combinations of polynomial order and number of groups for the analysis of frailty among participants without missing data of Fried frailty phenotype.
**Table S11:** Association of peak expiratory flow at baseline and its trajectories with frailty risk among participants without missing values of Fried frailty phenotype.

## Data Availability

Data are available to researchers on request for purposes of reproducing the results or replicating the procedure by directly contacting the corresponding author.
