# Supplementary material for: Peak Expiratory Flow and Its Trajectories Associated With Frailty in Older Adults: A Prospective Cohort Study
Source: J Cachexia Sarcopenia Muscle. 2025 Nov 20;16(6):e70123. doi: 10.1002/jcsm.70123 (PMC12641449; doi:10.1002/jcsm.70123)
Supplement: Supplementary file 1 — Figure S1: Selection flow of the study population for the association between peak expiratory flow at baseline and the risk of frailty in the HRS. Figure S2: Selection flow of the study population for the association between peak expiratory flow trajectories and the risk of frailty in the HRS. Figure S3: Subgroup analyses stratified by age and sex for the association between peak expiratory flow and the risk of frailty. Table S1: The criteria and cutoffs for Fried frailty phenotype, peak expiratory flow and covariates. Table S2: Diagnostics of models with all possible combinations of polynomial order and number of groups for the analysis of frailty. Table S3: Distribution characteristics of peak expiratory flow trajectories among the American population at baseline (n = 1826). Table S4: Association of peak expiratory flow at baseline with frailty in the Cox proportional hazard regression model for sensitivity analysis, HR (95% CI). Table S5: Diagnostics of models with all possible combinations of polynomial order and number of groups for the analysis of different frailty phenotypes. Table S6: Diagnostics of models with all possible combinations of polynomial order and number of groups for the analysis of frailty among never smokers. Table S7: Association of peak expiratory flow at baseline and its trajectories with frailty risk among never smokers. Table S8: Diagnostics of models with all possible combinations of polynomial order and number of groups for the analysis of frailty among participants without respiratory diseases at baseline. Table S9: Association of peak expiratory flow at baseline and its trajectories with frailty risk among participants without respiratory diseases at baseline. Table S10: Diagnostics of models with all possible combinations of polynomial order and number of groups for the analysis of frailty among participants without missing data of Fried frailty phenotype. Table S11: Association of peak expiratory flow at baseline and its trajectori [file JCSM-16-e70123-s001.docx]

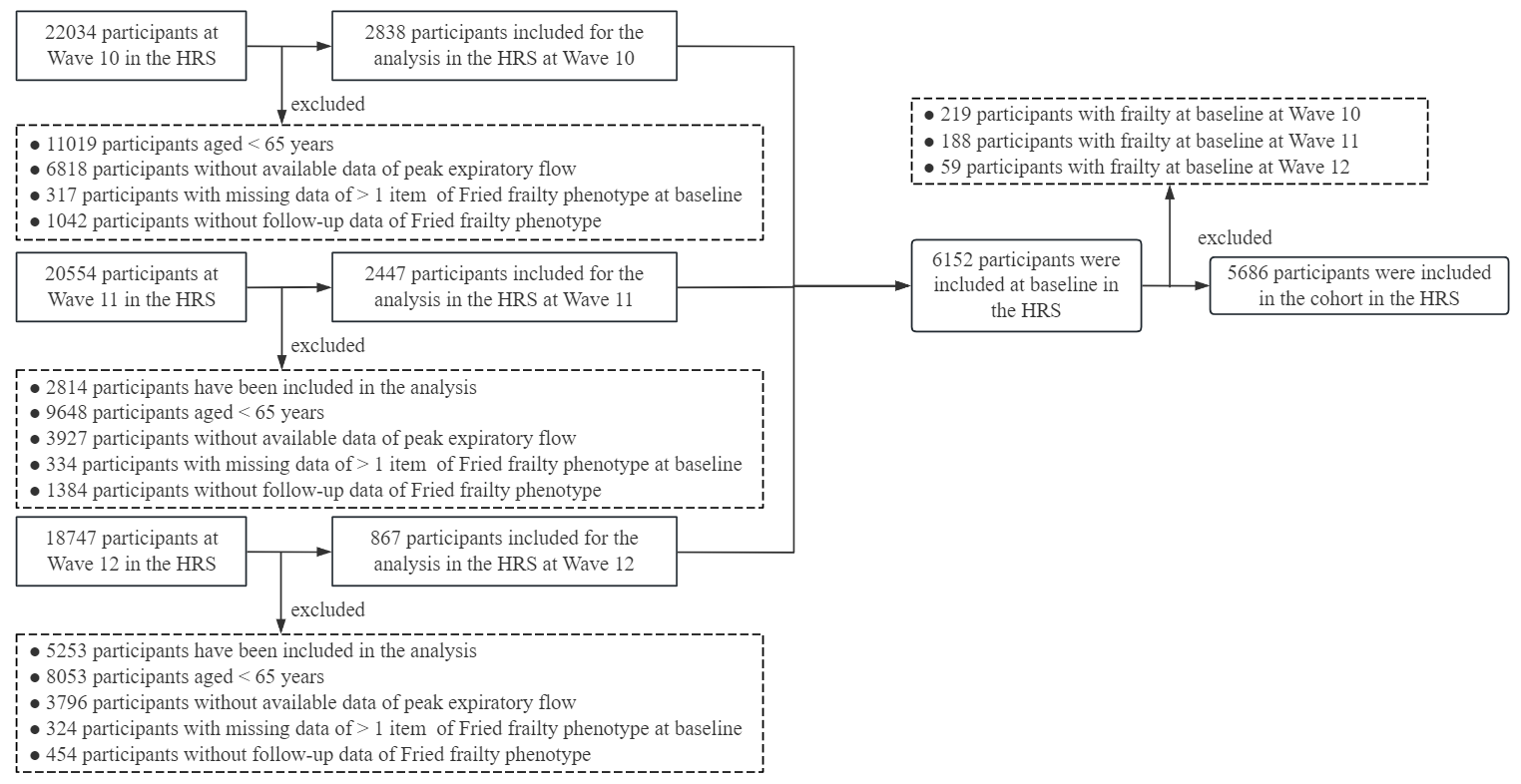


Notes: HRS: Health and Retirement Study

**Figure S1** Selection flow of the study population for the association between peak expiratory flow at baseline and the risk of frailty in the HRS.


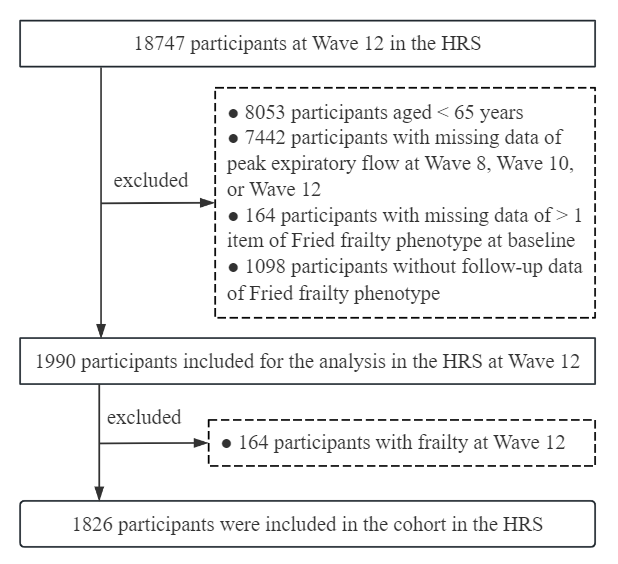


Notes: HRS: Health and Retirement Study

**Figure S2** Selection flow of the study population for the association between peak expiratory flow trajectories and the risk of frailty in the HRS.


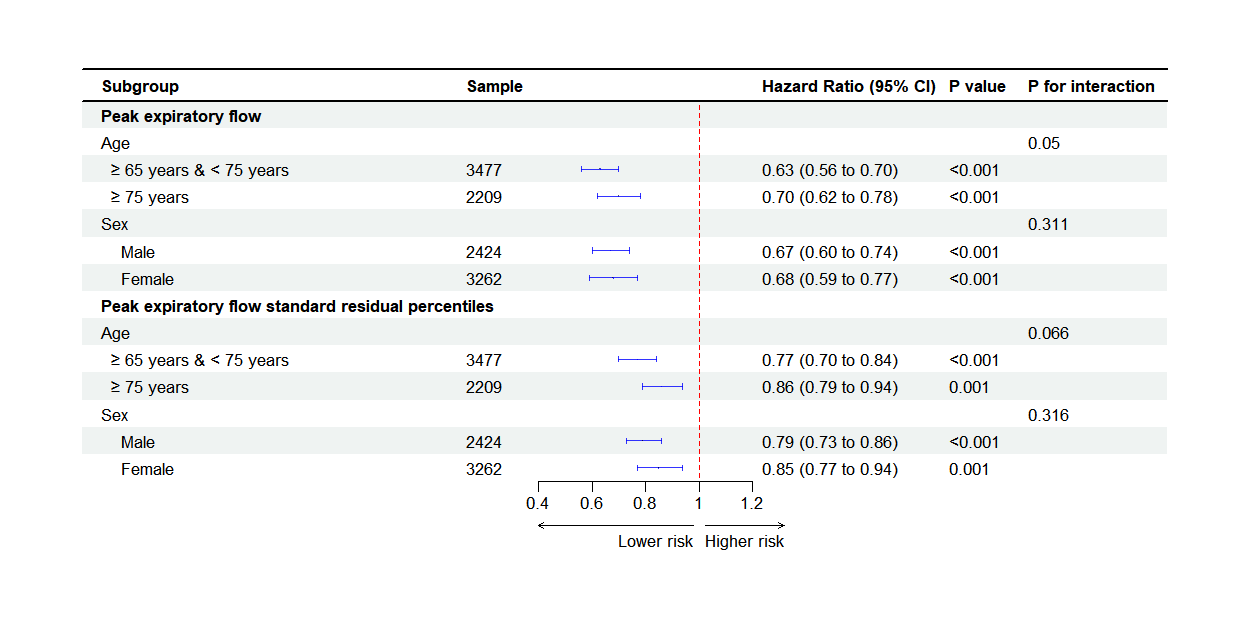


**Figure S3** Subgroup analyses stratified by age and sex for the association between peak expiratory flow and the risk of frailty.

**Table S1** The criteria and cutoffs for Fried frailty phenotype, peak expiratory flow, and covariates

| **Variables** | **Items** | **Cutoffs** |
| --- | --- | --- |
| Frailty |  |  |
| Slowness ^a^ | Gait speed was measured using a 2.5-m test. | 0 = No, 1 = Yes, defined as  **For men**  Gait speed ≤ 0.653 m/s for height ≤ 1.73 m  Gait speed ≤ 0.762 m/s for height > 1.73 m  **For women**  Gait speed ≤ 0.653 m/s for height ≤ 1.59 m  Gait speed ≤ 0.762 m/s for height > 1.59 m |
| Weakness ^a^ | We categorized weakness based on sex- and body mass index (BMI)- specific cutoffs of hand grip strength and slowness based on sex- and height-specific cutoffs of gait speed. | 0 = No, 1 = Yes, defined as  **For men**  Grip strength ≤ 29 kg for BMI ≤ 24 kg/m^2^  Grip strength ≤ 30 kg for 24 < BMI ≤ 26 kg/m^2^  Grip strength ≤ 32 kg for BMI > 26 kg/m^2^  **For women**  Grip strength ≤ 17 kg for BMI ≤ 23 kg/m^2^  Grip strength < 17.3 kg for 23 < BMI ≤ 26 kg/m^2^  Grip strength < 18 kg for 26 < BMI ≤ 29 kg/m^2^  Grip strength < 21 kg for BMI > 29 kg/m^2^ |
| Exhaustion | Exhaustion was based on reports of “could not get going” or “felt that everything was an effort” during the past week. | 0 = No, 1 = Yes, defined as self-report of yes to: (1) felt that everything I did was an effort or (2) could not get going in the last week |
| Inactivity | Participants met the criteria for inactivity if their total caloric expenditure of physical activities was in the lowest sex-specific 20%. Physical activity levels were quantified based on the frequency of engagement in vigorous, moderate, and light physical activities, which were assigned metabolic equivalent of task values of 8, 4, and 2, respectively. We derived a weighted-sum score for each participant, which represents the total energy expenditure associated with physical activities, taking into account both intensity and frequency. The weightings were determined by the frequency of physical activity engagement; daily, more than once a week, once a week, 1-3 times a month, and hardly ever or never were assigned scores of 7, 4, 1, 0.5, and 0, respectively[1]. | 0 = No, 1 = Yes, defined as total caloric expenditure in the lowest sex-specific 20%. |
| Shrinking ^a^ | Shrinking was based on the loss of body weight (4.5 kg or more within the past years). | 0 = No, 1 = Yes |
| Peak expiratory flow | Peak expiratory flow (PEF) was assessed utilizing a Mini-Wright peak flow meter (Clement Clarke International Ltd., Harlow, U.K.) coupled with a disposable mouthpiece, under the guidance of a trained interviewer. Three measurements were conducted with a 30-second interval between each, and the maximum value was adopted for the respondent’s PEF in our analyses. Participants were categorized into four groups based on the quartiles of measured PEF at baseline. | Q_1_ = 0, Q_2_ = 1, Q_3_ = 2, Q_4_ = 3, Q_5_ = 4 |
| Peak expiratory flow standardized residual | we operationalized the PEF variable through a two-step methodology. Initially, we established a baseline healthy subsample comprising individuals who had never smoked and were free from any chronic lung diseases, asthma, heart conditions, stroke, or cancer[2-3]. Predicted PEF values were derived from sex-specific predictive equations that incorporated age, stature, race, and ethnicity, as determined within this healthy subsample[2-3]. Subsequently, we calculated the standardized residual (SR) percentiles of PEF from the normalization of the ratio (measured PEF minus predicted PEF) divided by the standard deviation of the residuals, with SR = 0 corresponding to the 50th percentile[2-3]. | < 10th, ≥ 10th & < 30th, ≥ 30th & < 50th, ≥ 50th & < 80th, and ≥ 80th percentiles |
| Age |  | ≥ 65 and < 75 = 0, ≥ 75 = 1 |
| Sex |  | Male = 0, Female = 1 |
| Race |  | White = 0, Black = 1, Others = 2 |
| Place of residence | The place of residence was measured by the Beale codes provided in HRS’s Cross-Wave Census Region/Division and Mobility File, which are consolidated from the USDA’s Rural-Urban Continuum Codes. USDA’s full code list is available on their website. The crosswalk for HRS’s classifications is in the codebook of HRS’s Cross-Wave Census Region/Division and Mobility File. There are currently three sets of Beale codes provided: 1993, 2003, and 2013. For each wave of data, the Beale code with the closest year is used. If that value is missing, the next closest Beale code year is used. If that code is also missing, the furthest Beale code year is used. If the Beale code years are equidistant from the interview wave year, the most recent Beale code year is used first. | Urban = 0, Suburban = 1, Exurban (which also includes rural counties) = 2 |
| Educational level |  | High school or below = 0, Above high school = 1 |
| Marital status |  | Married, or partnered = 0, Separated, divorced, widowed, or never married = 1 |
| Per capita household income | Income measures are reported in nominal dollars. The HRS and AHEAD income components are summed to create the income measures found here in the RAND HRS Longitudinal File. For each derived income measure, a flag indicates whether any or all of its components were imputed. Individual income components and imputation flags are available in the RAND HRS Detailed Imputations File. Per capita household income refers to the total household income for the last calendar year divided by the total number of the household members. Participants were categorized into four groups based on the quartiles of per capita household income at baseline. | Q_1_ = 0, Q_2_ = 1, Q_3_ = 2, Q_4_ = 3 |
| Smoking status |  | Never smokers = 0, Former smokers = 1, Current smokers = 2 |
| Alcohol consumption |  | No = 0, Yes = 1 |
| Body mass index |  | < 25 = 0, ≥ 25 and < 30 = 1, ≥ 30 = 2 |
| Disability | Disability was assessed by the Activities of Daily Living (ADL), including bathing, dressing, eating, getting in/out of bed, walking across a room, and toileting. Individuals were considered disable if there was any difficulty with ADL[4]. | No = 0, Yes = 1 |
| Number of chronic diseases | In the HRS, chronic disease data were obtained by the question:“Has a doctor ever told you that you have any of the following conditions?” The conditions in question included hypertension, diabetes, cancer, heart diseases, stroke, arthritis, rheumatism, and emotional, nervous, or psychiatric problems. | 0 = 0, 1 = 1, ≥ 2 = 2 |
| Chronic respiratory disease status | Chronic respiratory disease status was determined based on the presence of chronic bronchitis, emphysema, asthma, or other chronic lung diseases and the receipt of associated treatment, which included medication, oxygen therapy, and physical or respiratory therapy. Participants were subsequently stratified into three distinct categories: no chronic respiratory diseases, chronic respiratory diseases with treatment, and chronic respiratory diseases without treatment. | No chronic respiratory diseases = 0, Chronic respiratory diseases with treatment = 1, Chronic respiratory diseases without treatment = 2 |
| Depression | Depression was assessed by the 8-item version of the Center for Epidemiologic Studies Depression Scale (CESD-8)[5]. | CESD-8 < 4 = 0, CESD-8 ≥ 4 = 1 |
| Cognitive function | Cognitive function was assessed with a composite score from tasks including immediate and delayed recall, serial sevens, and backward counting, classifying individuals into dementia (0-6), cognitive impairment without dementia (7-11), or normal cognition (12-27)[6]. | Normal cognition = 0, Cognitive impairment without dementia = 1, Dementia = 2 |
| Pain | Are you often troubled with pain? | No = 0, Yes = 1 |
| Systolic blood pressure | Trained interviewers used Omron HEM-780 Intellisense automated sphygmomanometers with ComFit cuffs to take systolic and diastolic readings from participants seated with both feet on the floor. Three sets of readings, taken between 45 and 60 seconds apart from a participant’s supported left arm with the palm facing upward, were averaged for the current analyses. Measured hypertension was defined based on mean sphygmomanometer readings and classified according to The Seventh Report of the Joint National Committee on Prevention, Detection, Evaluation, and Treatment of High Blood Pressure (JNC 7) criteria, the guideline during the period of the study: at least 140 mmHg for systolic blood pressure and/or at least 90 mmHg for diastolic blood pressure. | < 140 mmHg = 0, ≥ 140 mmHg = 1 |
| Diastolic blood pressure |  | < 90 mmHg = 0, ≥ 90 mmHg = 1 |
| C-reactive protein (CRP) | Blood chemistries were collected by HRS interviewers using a series of dried blood spots (DBS) that were placed on cards and shipped to either the University of Vermont or the University of Washington to be assayed. CRP cutpoints follow a joint report released by the American Heart Association and Centers for Disease Control and Prevention. Low HDL-C follows the American Heart Association (AHA) cutpoints for metabolic syndrome and TC levels were considered poor based on the definition from the AHA 2021 updated recommendations for cardiovascular health. HbA1c cutpoints follow glycemic goals established by the American Diabetes Association for diagnosis of diabetes. | ≤ 3 mg/L = 0, > 3 mg/L = 1 |
| High-density lipoprotein cholesterol (HDL) |  | Men: ≥ 40 mg/dL = 0, < 40 mg/dL = 1  Women: ≥ 50 mg/dL = 0, < 50 mg/dL = 1 |
| Total cholesterol (TC) |  | < 200 mg/dL = 0, ≥ 200 mg/dL = 1 |
| Glycosylated haemoglobin A1c (HbA1c) |  | < 6.5% = 0, ≥ 6.5% = 1 |
| Apolipoprotein E (APOE) gene | APOE genotype was assessed from saliva samples collected during home visits. A random half of the participants provided samples in 2006; the other half in 2008. Saliva-collection participation rates were 83% in 2006 and 84% in 2008. Genotyping was performed by the National Institute of Health Center for Inherited Disease Research, and then archived by the National Center for Biotechnology Information. | Non-ε4 carriers = 0, Heterozygous ɛ4 carriers = 1, Homozygous ɛ4 carriers = 2 |

Notes: ^a^: We utilized actual measurements for height and weight rather than relying on self-reported data.

**Table S2** Diagnostics of models with all possible combinations of polynomial order and number of groups for the analysis of frailty

| **Number of trajectories** | **Polynomial order ^a^** | **BIC (n = 5478) ^b^** | **BIC (n = 1826) ^c^** | **Proportion (%)** | **AvePP ^d^** | **OCC ^e^** | **Entropy** |
| --- | --- | --- | --- | --- | --- | --- | --- |
| 2 | 2 2 | -32837.98 | -32833.58 | 69.50, 30.50 | 0.97, 0.94 | 15.17, 38.50 | 0.878 |
| 3 | 2 2 2 | -32344.10 | -32337.51 | 49.84, 35.70, 14.46 | 0.94, 0.90, 0.93 | 16.58, 15.64, 84.19 | 0.842 |
| 4 | 2 2 2 2 | -32050.88 | -32042.09 | 19.44,44.63,25.69,10.24 | 0.88,0.90,0.91,0.95 | 30.82,11.58,30.50,161.14 | 0.838 |
| 5 | 2 2 2 2 2 | -31923.54 | -31912.55 | 12.98,41.46,24.15,14.78,6.63 | 0.90,0.89,0.87,0.90,0.93 | 60.08,10.95,20.18,49.39,192.98 | 0.828 |
| 5 | 1 1 1 1 1 | -31909.98 | -31901.74 | 13.25,41.62,23.93,14.73,6.46 | 0.89,0.88,0.87,0.89,0.94 | 54.17,10.79,21.07,48.77,213.33 | 0.827 |
| 5 | 1 1 1 1 2 | -31914.20 | -31905.41 | 13.25,41.62,23.93,14.68,6.52 | 0.89,0.88,0.87,0.90,0.93 | 54.12,10.78,21.05,49.67,200.32 | 0.827 |
| **5** | **1 1 1 2 1** | **-31906.81** | **-31898.02** | **13.09,41.29,24.15,14.84,6.63** | **0.90,0.89,0.86,0.90,0.93** | **57.25,11.14,20.09,48.93,195.75** | **0.827** |
| 5 | 1 1 1 2 2 | -31911.09 | -31901.76 | 13.09,41.29,24.15,14.84,6.63 | 0.90,0.89,0.86,0.89,0.93 | 57.22,11.13,20.08,48.90,196.47 | 0.827 |
| 5 | 1 1 2 1 1 | -31913.81 | -31905.02 | 13.25,41.68,23.93,14.68,6.46 | 0.89,0.88,0.87,0.89,0.94 | 54.32,10.72,21.21,49.20,209.31 | 0.827 |
| 5 | 1 1 2 1 2 | -31918.03 | -31908.69 | 13.25,41.68,23.93,14.68,6.46 | 0.89,0.88,0.87,0.89,0.94 | 54.28,10.72,21.20,49.11,210.50 | 0.827 |
| 5 | 1 1 2 2 1 | -31910.90 | -31901.56 | 13.14,41.29,24.15,14.79,6.63 | 0.89,0.89,0.87,0.90,0.93 | 56.03,11.14,20.20,49.52,192.76 | 0.828 |
| 5 | 1 1 2 2 2 | -31915.18 | -31905.29 | 13.09,41.29,24.21,14.79,6.63 | 0.90,0.89,0.86,0.90,0.93 | 57.26,11.13,19.98,49.49,193.43 | 0.827 |
| 5 | 1 2 1 1 1 | -31913.99 | -31905.20 | 13.14,41.73,24.04,14.62,6.46 | 0.90,0.88,0.87,0.90,0.94 | 56.76,10.69,20.64,50.64,212.43 | 0.827 |
| 5 | 1 2 1 1 2 | -31918.20 | -31908.87 | 13.14,41.73,24.04,14.57,6.52 | 0.90,0.88,0.87,0.90,0.93 | 56.76,10.68,20.61,51.60,199.66 | 0.827 |
| 5 | 1 2 1 2 1 | -31910.82 | -31901.48 | 12.98,41.51,24.04,14.84,6.63 | 0.90,0.89,0.87,0.89,0.93 | 60.16,10.87,20.49,48.79,195.00 | 0.828 |
| 5 | 1 2 1 2 2 | -31915.10 | -31905.22 | 12.98,41.51,24.04,14.84,6.63 | 0.90,0.89,0.87,0.89,0.93 | 60.12,10.87,20.48,48.75,195.72 | 0.828 |
| 5 | 1 2 2 1 1 | -31917.88 | -31908.55 | 13.14,41.73,23.99,14.68,6.46 | 0.90,0.88,0.87,0.89,0.94 | 56.88,10.70,20.99,49.10,208.63 | 0.827 |
| 5 | 1 2 2 1 2 | -31922.10 | -31912.21 | 13.14,41.73,23.99,14.68,6.46 | 0.90,0.88,0.87,0.89,0.94 | 56.84,10.69,20.97,49.01,209.86 | 0.827 |
| 5 | 1 2 2 2 1 | -31914.95 | -31905.07 | 12.98,41.51,24.10,14.79,6.63 | 0.90,0.89,0.87,0.90,0.93 | 60.17,10.87,20.39,49.42,192.30 | 0.828 |
| 5 | 1 2 2 2 2 | -31919.23 | -31908.80 | 12.98,41.51,24.10,14.79,6.63 | 0.90,0.89,0.87,0.90,0.93 | 60.13,10.86,20.38,49.38,192.98 | 0.828 |
| 5 | 2 1 1 1 1 | -31914.29 | -31905.50 | 13.25,41.62,23.93,14.73,6.46 | 0.89,0.88,0.87,0.89,0.94 | 54.16,10.79,21.07,48.77,213.33 | 0.827 |
| 5 | 2 1 1 1 2 | -31918.50 | -31909.16 | 13.25,41.62,23.93,14.68,6.52 | 0.89,0.88,0.87,0.90,0.93 | 54.12,10.79,21.05,49.67,200.34 | 0.827 |
| 5 | 2 1 1 2 1 | -31911.12 | -31901.78 | 13.09,41.29,24.15,14.84,6.63 | 0.90,0.89,0.86,0.90,0.93 | 57.24,11.14,20.09,48.93,195.76 | 0.827 |
| **5** | 2 1 1 2 2 | -31915.40 | -31905.51 | 13.09,41.29,24.15,14.84,6.63 | 0.90,0.89,0.86,0.89,0.93 | 57.18,11.13,20.08,48.89,196.37 | 0.827 |
| 5 | 2 1 2 1 1 | -31918.12 | -31908.78 | 13.25,41.68,23.93,14.68,6.46 | 0.89,0.88,0.87,0.89,0.94 | 54.31,10.72,21.22,49.20,209.21 | 0.827 |
| 5 | 2 1 2 1 2 | -31922.33 | -31912.44 | 13.25,41.68,23.93,14.68,6.46 | 0.89,0.88,0.87,0.89,0.94 | 54.27,10.72,21.20,49.11,210.49 | 0.827 |
| 5 | 2 1 2 2 1 | -31915.20 | -31905.32 | 13.14,41.29,24.15,14.79,6.63 | 0.89,0.89,0.87,0.90,0.93 | 56.02,11.14,20.20,49.52,192.75 | 0.828 |
| 5 | 2 1 2 2 2 | -31919.49 | -31909.05 | 13.09,41.29,24.21,14.79,6.63 | 0.90,0.89,0.86,0.90,0.93 | 57.25,11.13,19.99,49.49,193.45 | 0.827 |
| 5 | 2 2 1 1 1 | -31918.29 | -31908.95 | 13.20,41.68,24.04,14.62,6.46 | 0.89,0.89,0.87,0.90,0.94 | 55.47,10.77,20.64,50.65,212.50 | 0.827 |
| 5 | 2 2 1 1 2 | -31922.51 | -31912.62 | 13.14,41.73,24.04,14.57,6.52 | 0.90,0.88,0.87,0.90,0.93 | 56.68,10.69,20.62,51.60,199.55 | 0.827 |
| 5 | 2 2 1 2 1 | -31915.12 | -31905.24 | 12.98,41.51,24.04,14.84,6.63 | 0.90,0.89,0.87,0.89,0.93 | 60.12,10.87,20.49,48.80,195.05 | 0.828 |
| 5 | 2 2 1 2 2 | -31919.41 | -31908.97 | 12.92,41.57,24.04,14.84,6.63 | 0.90,0.88,0.87,0.89,0.93 | 61.49,10.79,20.49,48.76,195.65 | 0.828 |
| 5 | 2 2 2 1 1 | -31922.19 | -31912.30 | 13.14,41.73,23.99,14.68,6.46 | 0.90,0.88,0.87,0.89,0.94 | 56.84,10.70,20.99,49.11,208.73 | 0.827 |
| 5 | 2 2 2 1 2 | -31926.40 | -31915.96 | 13.14,41.73,23.99,14.68,6.46 | 0.90,0.88,0.87,0.89,0.94 | 56.80,10.69,20.98,49.02,209.93 | 0.827 |
| 5 | 2 2 2 2 1 | -31919.25 | -31908.82 | 12.98,41.51,24.10,14.79,6.63 | 0.90,0.89,0.87,0.90,0.93 | 60.13,10.87,20.39,49.43,192.28 | 0.828 |

Notes: When the polynomial order is three, the results were not statistically significant (P > 0.05). Although the linear term was not statistically significant, the linear term was retained according to previous studies.

^a^ Trajectory shapes; 1 = linear; 2 = quadratic; 3 = cubic; ^b^ BIC = Bayesian information criterion (for the total number of observations); ^c^ BIC = Bayesian information criterion (for the total number of participants); ^d^ Average group posterior probability. An AvePP greater than 0.7 for all groups is recommended; ^e^ Odds of correct classification. An OCC of 5 or more is recommended for all groups.

**Table S3** Distribution characteristics of peak expiratory flow trajectories among American population at baseline (n = 1826)

| Outcomes/ Covariates | Peak expiratory flow trajectories | | | | |
| --- | --- | --- | --- | --- | --- |
|  | Low  n (%) | Lower-moderate  n (%) | Moderate  n (%) | Upper-moderate  n(%) | High  n(%) |
| Sample | 239 | 754 | 441 | 271 | 121 |
| Age |  |  |  |  |  |
| ≥ 65 years & < 75 years | 90 (37.66) | 386 (51.19) | 267 (60.54) | 164 (60.52) | 86 (71.07) |
| ≥ 75 years | 149 (62.34) | 368 (48.81) | 174 (39.46) | 107 (39.48) | 35 (28.93) |
| Sex |  |  |  |  |  |
| Male | 16 (6.69) | 108 (14.32) | 241 (54.65) | 263 (97.05) | 121 (100.00) |
| Female | 223 (93.31) | 646 (85.68) | 200 (45.35) | 8 (2.95) | 0 (0.00) |
| Race |  |  |  |  |  |
| White | 186 (77.82) | 634 (84.08) | 366 (82.99) | 246 (90.77) | 111 (91.73) |
| Black | 44 (18.41) | 87 (11.54) | 57 (12.93) | 20 (7.38) | 4 (3.31) |
| Others | 9 (3.77) | 33 (4.38) | 18 (4.08) | 5 (1.85) | 6 (4.96) |
| Place of residence |  |  |  |  |  |
| Urban | 117 (48.95) | 358 (47.48) | 212 (48.07) | 144 (53.13) | 56 (46.28) |
| Suburban | 50 (20.92) | 190 (25.20) | 107 (24.26) | 64 (23.62) | 34 (28.10) |
| Exurban | 72 (30.13) | 206 (27.32) | 122 (27.66) | 63 (23.25) | 31 (25.62) |
| Educational level |  |  |  |  |  |
| High school or below | 152 (63.60) | 378 (50.13) | 176 (39.91) | 90 (33.21) | 42 (34.71) |
| Above high school | 87 (36.40) | 376 (49.87) | 265 (60.09) | 181 (66.79) | 79 (65.29) |
| Marital status |  |  |  |  |  |
| Married or partnered | 108 (45.19) | 428 (56.76) | 327 (74.15) | 234 (86.35) | 98 (80.99) |
| Others | 131 (54.81) | 326 (43.24) | 114 (25.85) | 37 (13.65) | 23 (19.01) |
| Per capita household income ($) |  |  |  |  |  |
| Q_1_ | 101 (42.26) | 201 (26.66) | 98 (22.22) | 44 (16.24) | 12 (9.92) |
| Q_2_ | 61 (25.52) | 205 (27.18) | 101 (22.90) | 58 (21.40) | 32 (26.45) |
| Q_3_ | 46 (19.25) | 174 (23.08) | 120 (27.22) | 82 (30.26) | 33 (27.27) |
| Q_4_ | 31 (12.97) | 174 (23.08) | 122 (27.66) | 87 (32.10) | 44 (36.36) |
| Smoking status |  |  |  |  |  |
| Never | 116 (48.54) | 396 (52.52) | 193 (43.76) | 112 (41.33) | 49 (40.50) |
| Former | 98 (41.00) | 309 (40.98) | 228 (51.70) | 140 (51.66) | 65 (53.71) |
| Current | 25 (10.46) | 49 (6.50) | 20 (4.54) | 19 (7.01) | 7 (5.79) |
| Alcohol consumption |  |  |  |  |  |
| No | 136 (56.90) | 339 (44.96) | 166 (37.64) | 85 (31.37) | 29 (23.97) |
| Yes | 103 (43.10) | 415 (55.04) | 275 (62.36) | 186 (68.63) | 92 (76.03) |
| Body mass index (kg/m^2^) |  |  |  |  |  |
| < 25 | 65 (27.20) | 170 (22.55) | 74 (16.78) | 39 (14.39) | 8 (6.61) |
| ≥ 25 & < 30 | 95 (39.75) | 272 (36.07) | 168 (38.10) | 121 (44.65) | 67 (55.37) |
| ≥ 30 | 79 (33.05) | 312 (41.38) | 199 (45.12) | 111 (40.96) | 46 (38.02) |
| Disability |  |  |  |  |  |
| No | 209 (87.45) | 674 (89.39) | 390 (88.44) | 252 (92.99) | 110 (90.91) |
| Yes | 30 (12.55) | 80 (10.61) | 51 (11.56) | 19 (7.01) | 11 (9.09) |
| Number of chronic diseases |  |  |  |  |  |
| 0 | 10 (4.18) | 56 (7.43) | 44 (9.98) | 23 (8.49) | 19 (15.71) |
| 1 | 39 (16.32) | 151 (20.03) | 111 (25.17) | 62 (22.88) | 41 (33.88) |
| ≥ 2 | 190 (79.50) | 547 (72.54) | 286 (64.85) | 186 (68.63) | 61 (50.41) |
| Chronic respiratory disease status |  |  |  |  |  |
| No chronic respiratory diseases | 183 (76.57) | 684 (90.72) | 422 (95.69) | 261 (96.31) | 120 (99.17) |
| Chronic respiratory diseases with treatment | 40 (16.74) | 43 (5.70) | 9 (2.04) | 3 (1.11) | 1 (0.83) |
| Chronic respiratory diseases without treatment | 16 (6.69) | 27 (3.58) | 10 (2.27) | 7 (2.58) | 0 (0.00) |
| Depression |  |  |  |  |  |
| No | 207 (86.61) | 690 (91.51) | 417 (94.56) | 261 (96.31) | 115 (95.04) |
| Yes | 32 (13.39) | 64 (8.49) | 24 (5.44) | 10 (3.69) | 6 (4.96) |
| Cognitive function |  |  |  |  |  |
| Normal | 185 (77.41) | 640 (84.88) | 390 (88.44) | 252 (92.99) | 114 (94.21) |
| Mild cognitive impairment | 43 (17.99) | 102 (13.53) | 46 (10.43) | 16 (5.90) | 7 (5.79) |
| Dementia | 11 (4.60) | 12 (1.59) | 5 (1.13) | 3 (1.11) | 0 (0.00) |
| Pain |  |  |  |  |  |
| No | 147 (61.51) | 484 (64.19) | 299 (67.80) | 190 (70.11) | 93 (76.86) |
| Yes | 92 (38.49) | 270 (35.81) | 142 (32.20) | 81 (29.89) | 28 (23.14) |
| Systolic blood pressure (mmHg) |  |  |  |  |  |
| < 140 | 161 (67.36) | 544 (72.15) | 312 (70.75) | 189 (69.74) | 90 (74.38) |
| ≥ 140 | 78 (32.64) | 210 (27.85) | 129 (29.25) | 82 (30.26) | 31 (25.62) |
| Diastolic blood pressure (mmHg) |  |  |  |  |  |
| < 90 | 206 (86.19) | 676 (89.66) | 386 (87.53) | 247 (91.14) | 109 (90.08) |
| ≥ 90 | 33 (13.81) | 78 (10.34) | 55 (12.47) | 24 (8.86) | 12 (9.92) |
| High-density lipoprotein cholesterol (mg/L) |  |  |  |  |  |
| < 50 for males / < 40 for females | 205 (85.77) | 623 (82.63) | 300 (68.03) | 135 (49.82) | 50 (41.32) |
| ≥ 50 for males / ≥ 40 for females | 34 (14.23) | 131 (17.37) | 141 (31.97) | 136 (50.18) | 71 (58.68) |
| Total cholesterol (mg/L) |  |  |  |  |  |
| < 200 | 141 (59.00) | 399 (52.92) | 261 (59.18) | 183 (67.53) | 77 (63.64) |
| ≥ 200 | 98 (41.00) | 355 (47.08) | 180 (40.82) | 88 (32.47) | 44 (36.36) |
| C-reactive protein (mg/L) |  |  |  |  |  |
| < 3 | 161 (67.36) | 499 (66.18) | 304 (68.93) | 203 (74.91) | 93 (76.86) |
| ≥ 3 | 78 (32.64) | 255 (33.82) | 137 (31.07) | 68 (25.09) | 28 (23.14) |
| Glycosylated haemoglobin A1c (%) |  |  |  |  |  |
| < 6.5 | 196 (82.01) | 640 (84.88) | 368 (83.45) | 231 (85.24) | 111 (91.74) |
| ≥ 6.5 | 43 (17.99) | 114 (15.12) | 73 (16.55) | 40 (14.76) | 10 (8.26) |
| Apolipoprotein E gene |  |  |  |  |  |
| Non-ε4 carriers | 184 (76.99) | 572 (75.86) | 334 (75.73) | 221 (81.55) | 94 (77.69) |
| Heterozygous ɛ4 carriers | 54 (22.59) | 169 (22.41) | 97 (22.00) | 49 (18.08) | 25 (20.66) |
| Homozygous ɛ4 carriers | 1 (0.42) | 13 (1.73) | 10 (2.27) | 1 (0.37) | 2 (1.65) |
| Frailty |  |  |  |  |  |
| Robust | 65 (27.20) | 352 (46.68) | 224 (50.79) | 161 (59.41) | 87 (71.90) |
| Pre-frail | 174 (72.80) | 402 (53.32) | 217 (49.21) | 110 (40.59) | 34 (28.10) |

Notes: IQR: interquartile range

**Table S4** Association of peak expiratory flow at baseline with frailty in the Cox proportional hazard regression model for sensitivity analysis, HR (95%CI)

| Peak expiratory flow | Robustness (n = 2712) ^a^ | Pre-frailty (n = 2974) ^b^ |
| --- | --- | --- |
| Measured values at baseline |  |  |
| Q_1_ | **2.84 (1.78-4.53)** | **1.59 (1.25-2.01)** |
| Q_2_ | **1.70 (1.05-2.74)** | **1.55 (1.23-1.95)** |
| Q_3_ | 1.54 (0.95-2.48) | **1.31 (1.03-1.66)** |
| Q_4_ | 1.30 (0.78-2.16) | 1.09 (0.86-1.37) |
| Q_5_ | Reference | Reference |
| Standard residual percentiles at baseline |  |  |
| ≥ 80th | Reference | Reference |
| ≥ 50th & < 80th | 1.22 (0.79-1.90) | 1.04 (0.93-1.17) |
| ≥ 30th & < 50th | 1.33 (0.83-2.15) | **1.22 (1.08-1.38)** |
| ≥ 10th & < 30th | **1.98 (1.27-3.08)** | **1.30 (1.15-1.48)** |
| < 10th | **3.10 (1.92-5.00)** | **1.78 (1.52-2.07)** |

Notes: Boldface: *P* < 0.05; HR: hazard ratio; CI: confidence interval; ^a^: hazard ratio after adjusting for age, educational level, per capita household income, alcohol consumption, body mass index, disability, number of chronic diseases, cognitive function, pain, and glycosylated haemoglobin A1c; ^b^: hazard ratio after adjusting for age, educational level, marital status, per capita household income, alcohol consumption, body mass index, disability, number of chronic diseases, chronic respiratory disease status, depression, cognitive function, pain, total cholesterol, C-reactive protein, and glycosylated haemoglobin A1c.

**Table S5** Diagnostics of models with all possible combinations of polynomial order and number of groups for the analysis of different frailty phenotypes

| **Number of trajectories** | **Polynomial order ^a^** | **BIC (n = 2667) ^b^** | **BIC (n = 889) ^c^** | **Proportion (%)** | **AvePP ^d^** | **OCC ^e^** | **Entropy** |
| --- | --- | --- | --- | --- | --- | --- | --- |
| 2 | 2 2 | -15952.91 | -15948.52 | 66.25,33.75 | 0.98,0.95 | 22.14,39.77 | 0.893 |
| 3 | 2 2 2 | -15693.23 | -15686.63 | 50.28,34.42,15.30 | 0.96,0.93,0.95 | 25.40,23.54,102.36 | 0.881 |
| 4 | 2 2 2 2 | -15585.12 | -15576.33 | 33.41,34.42,21.71,10.46 | 0.92,0.87,0.92,0.95 | 22.23,12.66,38.99,162.70 | 0.835 |
| 5 | 2 2 2 2 2 | -15510.01 | -15499.02 | 6.97,40.38,24.86,18.00,9.79 | 0.92,0.91,0.88,0.92,0.94 | 146.42,14.50,22.39,52.75,148.89 | 0.849 |
| 5 | 1 1 1 1 1 | -15496.36 | -15488.12 | 6.97,40.61,24.75,17.89,9.79 | 0.92,0.90,0.88,0.92,0.94 | 153.46,13.67,22.53,54.31,137.79 | 0.847 |
| 5 | 1 1 1 1 2 | -15500.30 | -15491.51 | 6.97,40.61,24.75,17.89,9.79 | 0.92,0.90,0.88,0.92,0.94 | 153.47,13.67,22.53,54.39,137.97 | 0.847 |
| **5** | **1 1 1 2 1** | **-15496.07** | **-15487.28** | **6.97,40.61,24.75,17.89,9.79** | **0.92,0.90,0.88,0.92,0.94** | **155.71,13.67,22.50,54.56,149.69** | **0.848** |
| 5 | 1 1 1 2 2 | -15499.97 | -15490.63 | 6.97,40.61,24.75,17.89,9.79 | 0.92,0.90,0.88,0.92,0.94 | 155.83,13.68,22.52,54.66,149.22 | 0.848 |
| 5 | 1 1 2 1 1 | -15499.40 | -15490.61 | 6.97,40.38,24.86,18.00,9.79 | 0.92,0.91,0.88,0.92,0.94 | 153.71,14.12,22.13,51.75,137.44 | 0.847 |
| 5 | 1 1 2 1 2 | -15503.34 | -15494.00 | 6.97,40.38,24.86,18.00,9.79 | 0.92,0.91,0.88,0.92,0.94 | 153.73,14.12,22.13,51.82,137.62 | 0.847 |
| 5 | 1 1 2 2 1 | -15499.42 | -15490.08 | 6.97,40.49,24.86,17.89,9.79 | 0.92,0.90,0.88,0.92,0.94 | 155.44,13.87,22.18,54.68,148.40 | 0.848 |
| 5 | 1 1 2 2 2 | -15503.32 | -15493.43 | 6.97,40.49,24.86,17.89,9.79 | 0.92,0.90,0.88,0.92,0.94 | 155.56,13.88,22.21,54.75,147.87 | 0.848 |
| 5 | 1 2 1 1 1 | -15500.08 | -15491.29 | 6.97,40.61,24.75,17.89,9.79 | 0.92,0.90,0.88,0.92,0.94 | 154.95,13.68,22.48,54.13,137.42 | 0.847 |
| 5 | 1 2 1 1 2 | -15504.02 | -15494.68 | 6.97,40.61,24.75,17.89,9.79 | 0.92,0.90,0.88,0.92,0.94 | 154.97,13.68,22.48,54.21,137.60 | 0.847 |
| 5 | 1 2 1 2 1 | -15499.79 | -15490.45 | 6.97,40.61,24.75,17.89,9.79 | 0.92,0.90,0.88,0.92,0.94 | 157.24,13.68,22.45,54.37,149.29 | 0.848 |
| 5 | 1 2 1 2 2 | -15503.69 | -15493.80 | 6.97,40.61,24.75,17.89,9.79 | 0.92,0.90,0.88,0.92,0.94 | 157.37,13.70,22.48,54.44,148.71 | 0.848 |
| 5 | 1 2 2 1 1 | -15503.17 | -15493.83 | 6.97,40.61,24.63,18.11,9.67 | 0.92,0.90,0.88,0.92,0.94 | 155.03,13.65,23.12,49.45,151.80 | 0.847 |
| 5 | 1 2 2 1 2 | -15507.11 | -15497.22 | 6.97,40.61,24.63,18.00,9.79 | 0.92,0.90,0.88,0.92,0.94 | 155.05,13.65,23.12,51.63,137.21 | 0.848 |
| 5 | 1 2 2 2 1 | -15503.18 | -15493.29 | 6.97,40.61,24.75,17.89,9.79 | 0.92,0.90,0.88,0.92,0.94 | 156.84,13.65,22.65,54.48,148.00 | 0.848 |
| 5 | 1 2 2 2 2 | -15507.08 | -15496.64 | 6.97,40.61,24.75,17.89,9.79 | 0.92,0.90,0.88,0.92,0.94 | 156.97,13.66,22.68,54.56,147.51 | 0.848 |
| 5 | 2 1 1 1 1 | -15499.24 | -15490.45 | 6.97,40.38,24.86,18.00,9.79 | 0.91,0.91,0.88,0.92,0.94 | 143.28,14.49,22.24,52.48,138.95 | 0.848 |
| 5 | 2 1 1 1 2 | -15503.18 | -15493.84 | 6.97,40.38,24.86,18.00,9.79 | 0.91,0.91,0.88,0.92,0.94 | 143.32,14.49,22.24,52.55,139.12 | 0.849 |
| 5 | 2 1 1 2 1 | -15498.95 | -15489.61 | 6.97,40.38,24.97,17.89,9.79 | 0.92,0.91,0.88,0.92,0.94 | 145.30,14.50,21.72,55.08,150.98 | 0.849 |
| 5 | 2 1 1 2 2 | -15502.85 | -15492.96 | 6.97,40.38,24.97,17.89,9.79 | 0.92,0.91,0.88,0.92,0.94 | 145.31,14.51,21.74,55.23,150.62 | 0.848 |
| 5 | 2 1 2 1 1 | -15502.28 | -15492.94 | 6.97,40.38,24.86,18.00,9.79 | 0.91,0.91,0.88,0.92,0.94 | 143.52,14.46,22.36,52.24,138.58 | 0.848 |
| 5 | 2 1 2 1 2 | -15506.21 | -15496.33 | 6.97,40.27,24.97,18.00,9.79 | 0.91,0.91,0.88,0.92,0.94 | 143.36,14.73,21.87,52.34,138.82 | 0.849 |
| 5 | 2 1 2 2 1 | -15502.29 | -15492.40 | 6.97,40.27,24.97,18.00,9.79 | 0.92,0.91,0.88,0.92,0.94 | 144.93,14.73,21.92,52.83,149.71 | 0.849 |
| 5 | 2 1 2 2 2 | -15506.19 | -15495.76 | 6.97,40.27,24.97,18.00,9.79 | 0.92,0.91,0.88,0.92,0.94 | 145.35,14.72,21.93,52.90,149.18 | 0.848 |
| 5 | 2 2 1 1 1 | -15503.02 | -15493.68 | 6.97,40.61,24.63,18.00,9.79 | 0.92,0.91,0.88,0.92,0.94 | 145.01,14.00,23.23,52.30,138.53 | 0.848 |
| 5 | 2 2 1 1 2 | -15506.96 | -15497.07 | 6.97,40.61,24.63,18.00,9.79 | 0.92,0.91,0.88,0.92,0.94 | 144.71,14.01,23.23,52.39,138.79 | 0.849 |
| 5 | 2 2 1 2 1 | -15502.72 | -15492.84 | 6.97,40.61,24.75,17.89,9.79 | 0.92,0.91,0.88,0.92,0.94 | 146.71,14.01,22.68,54.93,150.59 | 0.849 |
| 5 | 2 2 1 2 2 | -15506.62 | -15496.19 | 6.97,40.61,24.75,17.89,9.79 | 0.92,0.91,0.88,0.92,0.94 | 146.86,14.02,22.70,55.02,150.13 | 0.848 |
| 5 | 2 2 2 1 1 | -15506.10 | -15496.21 | 6.97,40.38,24.86,18.00,9.79 | 0.92,0.91,0.88,0.92,0.94 | 144.73,14.49,22.32,52.08,138.19 | 0.848 |
| 5 | 2 2 2 1 2 | -15510.03 | -15499.60 | 6.97,40.38,24.86,18.00,9.79 | 0.92,0.91,0.88,0.92,0.94 | 144.55,14.50,22.33,52.17,138.46 | 0.849 |
| 5 | 2 2 2 2 1 | -15506.10 | -15495.67 | 6.97,40.38,24.86,18.00,9.79 | 0.92,0.91,0.88,0.92,0.94 | 146.39,14.49,22.36,52.64,149.33 | 0.849 |

Notes: When the polynomial order is three, the results were not statistically significant (P > 0.05). Although the linear term was not statistically significant, the linear term was retained according to previous studies.

^a^ Trajectory shapes; 1 = linear; 2 = quadratic; 3 = cubic; ^b^ BIC = Bayesian information criterion (for the total number of observations); ^c^ BIC = Bayesian information criterion (for the total number of participants); ^d^ Average group posterior probability. An AvePP greater than 0.7 for all groups is recommended; ^e^ Odds of correct classification. An OCC of 5 or more is recommended for all groups.

**Table S6** Diagnostics of models with all possible combinations of polynomial order and number of groups for the analysis of frailty among never smokers

| **Number of trajectories** | **Polynomial order ^a^** | **BIC (n = 2598) ^b^** | **BIC (n = 866) ^c^** | **Proportion (%)** | **AvePP ^d^** | **OCC ^e^** | **Entropy** |
| --- | --- | --- | --- | --- | --- | --- | --- |
| 2 | 2 2 | -15457.12 | -15452.73 | 76.10,23.90 | 0.98,0.96 | 14.55,75.61 | 0.910 |
| 3 | 2 2 2 | -15244.52 | -15237.93 | 44.34,38.80,16.86 | 0.91,0.89,0.95 | 13.30,12.79,90.44 | 0.812 |
| 4 | 2 2 2 2 | -15115.27 | -15106.48 | 19.40,51.27,20.44,8.89 | 0.89,0.90,0.91,0.95 | 34.37,8.96,39.22,184.91 | 0.840 |
| 5 | 2 2 2 2 2 | -15043.24 | -15032.25 | 11.78,42.84,24.71,14.32,6.35 | 0.89,0.89,0.87,0.92,0.94 | 58.95,10.62,19.85,65.10,245.92 | 0.831 |
| 5 | 1 1 1 1 1 | -15026.17 | -15017.93 | 11.78,42.96,24.48,14.43,6.35 | 0.89,0.89,0.87,0.91,0.94 | 59.11,10.47,20.50,60.69,234.78 | 0.830 |
| 5 | 1 1 1 1 2 | -15029.64 | -15020.85 | 11.78,43.07,24.36,14.43,6.35 | 0.89,0.89,0.87,0.91,0.94 | 59.33,10.35,20.96,60.40,222.55 | 0.830 |
| **5** | **1 1 1 2 1** | **-15028.02** | **-15019.23** | **11.78,42.96,24.60,14.32,6.35** | **0.89,0.89,0.87,0.92,0.94** | **59.18,10.44,20.28,64.66,251.09** | **0.831** |
| 5 | 1 1 1 2 2 | -15031.58 | -15022.25 | 11.78,42.96,24.60,14.32,6.35 | 0.89,0.89,0.87,0.92,0.94 | 59.28,10.45,20.28,64.60,244.44 | 0.830 |
| 5 | 1 1 2 1 1 | -15030.05 | -15021.26 | 11.78,42.96,24.48,14.43,6.35 | 0.89,0.89,0.87,0.91,0.94 | 59.18,10.47,20.46,60.87,235.24 | 0.831 |
| 5 | 1 1 2 1 2 | -15033.52 | -15024.18 | 11.78,42.96,24.48,14.43,6.35 | 0.89,0.89,0.87,0.91,0.94 | 59.41,10.50,20.46,60.57,223.01 | 0.831 |
| 5 | 1 1 2 2 1 | -15031.84 | -15022.50 | 11.78,42.84,24.71,14.32,6.35 | 0.89,0.89,0.87,0.92,0.94 | 59.35,10.61,19.79,65.04,252.26 | 0.831 |
| 5 | 1 1 2 2 2 | -15035.41 | -15025.53 | 11.78,42.84,24.71,14.32,6.35 | 0.89,0.89,0.87,0.92,0.94 | 59.45,10.62,19.80,64.98,245.71 | 0.830 |
| 5 | 1 2 1 1 1 | -15030.10 | -15021.31 | 11.78,42.96,24.48,14.43,6.35 | 0.89,0.89,0.87,0.91,0.94 | 59.10,10.47,20.50,60.69,234.78 | 0.830 |
| 5 | 1 2 1 1 2 | -15033.57 | -15024.23 | 11.78,43.07,24.36,14.43,6.35 | 0.89,0.89,0.87,0.91,0.94 | 59.33,10.35,20.96,60.40,222.57 | 0.831 |
| 5 | 1 2 1 2 1 | -15031.95 | -15022.61 | 11.78,42.96,24.60,14.32,6.35 | 0.89,0.89,0.87,0.92,0.94 | 59.18,10.44,20.28,64.66,251.09 | 0.831 |
| 5 | 1 2 1 2 2 | -15035.52 | -15025.63 | 11.78,42.96,24.60,14.32,6.35 | 0.89,0.89,0.87,0.92,0.94 | 59.28,10.45,20.28,64.60,244.44 | 0.830 |
| 5 | 1 2 2 1 1 | -15033.98 | -15024.65 | 11.78,42.96,24.48,14.43,6.35 | 0.89,0.89,0.87,0.91,0.94 | 59.17,10.47,20.46,60.86,235.23 | 0.830 |
| 5 | 1 2 2 1 2 | -15037.45 | -15027.56 | 11.78,42.96,24.48,14.43,6.35 | 0.89,0.89,0.87,0.91,0.94 | 59.40,10.51,20.46,60.57,223.01 | 0.831 |
| 5 | 1 2 2 2 1 | -15035.77 | -15025.89 | 11.78,42.84,24.71,14.32,6.35 | 0.89,0.89,0.87,0.92,0.94 | 59.35,10.61,19.79,65.03,252.25 | 0.831 |
| 5 | 1 2 2 2 2 | -15039.34 | -15028.91 | 11.78,42.84,24.71,14.32,6.35 | 0.89,0.89,0.87,0.92,0.94 | 59.44,10.62,19.79,64.98,245.72 | 0.830 |
| 5 | 2 1 1 1 1 | -15030.07 | -15021.28 | 11.78,42.96,24.48,14.43,6.35 | 0.89,0.89,0.87,0.91,0.94 | 58.69,10.48,20.55,60.77,235.08 | 0.830 |
| 5 | 2 1 1 1 2 | -15033.54 | -15024.20 | 11.78,43.07,24.36,14.43,6.35 | 0.89,0.89,0.87,0.91,0.94 | 58.91,10.35,21.00,60.47,222.85 | 0.831 |
| 5 | 2 1 1 2 1 | -15031.91 | -15022.58 | 11.78,42.96,24.60,14.32,6.35 | 0.89,0.89,0.87,0.92,0.94 | 58.76,10.44,20.32,64.74,251.39 | 0.831 |
| 5 | 2 1 1 2 2 | -15035.48 | -15025.59 | 11.78,42.96,24.60,14.32,6.35 | 0.89,0.89,0.87,0.92,0.94 | 58.86,10.46,20.32,64.68,244.75 | 0.830 |
| 5 | 2 1 2 1 1 | -15033.95 | -15024.61 | 11.78,42.96,24.48,14.43,6.35 | 0.89,0.89,0.87,0.91,0.94 | 58.76,10.48,20.51,60.94,235.54 | 0.830 |
| 5 | 2 1 2 1 2 | -15037.42 | -15027.53 | 11.78,42.96,24.48,14.43,6.35 | 0.89,0.89,0.87,0.91,0.94 | 58.99,10.51,20.51,60.65,223.31 | 0.831 |
| 5 | 2 1 2 2 1 | -15035.74 | -15025.85 | 11.78,42.84,24.71,14.32,6.35 | 0.89,0.89,0.87,0.92,0.94 | 58.93,10.61,19.84,65.11,252.56 | 0.831 |
| 5 | 2 1 2 2 2 | -15039.31 | -15028.87 | 11.78,42.84,24.71,14.32,6.35 | 0.89,0.89,0.87,0.92,0.94 | 59.03,10.63,19.84,65.06,246.01 | 0.830 |
| 5 | 2 2 1 1 1 | -15034.00 | -15024.66 | 11.78,42.96,24.48,14.43,6.35 | 0.89,0.89,0.87,0.91,0.94 | 58.67,10.48,20.55,60.76,235.07 | 0.830 |
| 5 | 2 2 1 1 2 | -15037.47 | -15027.58 | 11.78,42.96,24.48,14.43,6.35 | 0.89,0.89,0.87,0.91,0.94 | 58.90,10.51,20.55,60.47,222.84 | 0.831 |
| 5 | 2 2 1 2 1 | -15035.84 | -15025.96 | 11.78,42.96,24.60,14.32,6.35 | 0.89,0.89,0.87,0.92,0.94 | 58.74,10.44,20.32,64.74,251.38 | 0.831 |
| 5 | 2 2 1 2 2 | -15039.41 | -15028.98 | 11.78,42.96,24.60,14.32,6.35 | 0.89,0.89,0.87,0.92,0.94 | 58.84,10.46,20.32,64.68,244.74 | 0.830 |
| 5 | 2 2 2 1 1 | -15037.88 | -15027.99 | 11.78,42.96,24.48,14.43,6.35 | 0.89,0.89,0.87,0.91,0.94 | 58.74,10.48,20.51,60.94,235.54 | 0.830 |
| 5 | 2 2 2 1 2 | -15041.35 | -15030.91 | 11.78,42.96,24.48,14.43,6.35 | 0.89,0.89,0.87,0.91,0.94 | 58.96,10.51,20.51,60.65,223.32 | 0.831 |
| 5 | 2 2 2 2 1 | -15039.67 | -15029.23 | 11.78,42.84,24.71,14.32,6.35 | 0.89,0.89,0.87,0.92,0.94 | 58.90,10.62,19.83,65.11,252.56 | 0.831 |

Notes: When the polynomial order is three, the results were not statistically significant (P > 0.05). Although the linear term was not statistically significant, the linear term was retained according to previous studies.

^a^ Trajectory shapes; 1 = linear; 2 = quadratic; 3 = cubic; ^b^ BIC = Bayesian information criterion (for the total number of observations); ^c^ BIC = Bayesian information criterion (for the total number of participants); ^d^ Average group posterior probability. An AvePP greater than 0.7 for all groups is recommended; ^e^ Odds of correct classification. An OCC of 5 or more is recommended for all groups.

**Table S7** Association of peak expiratory flow at baseline and its trajectories with frailty risk among never smokers

| Peak expiratory flow | HR (95%CI) |
| --- | --- |
| Measured values at baseline (n = 2606) |  |
| Q_1_ | **3.38 (2.22-5.13)** |
| Q_2_ | **2.53 (1.66-3.84)** |
| Q_3_ | **2.95 (1.98-4.40)** |
| Q_4_ | **2.02 (1.35-3.02)** |
| Q_5_ | Reference |
| Standard residual percentiles at baseline (n = 2606) |  |
| ≥ 80th | Reference |
| ≥ 50th & < 80th | 1.33 (0.99-1.78) |
| ≥ 30th & < 50th | 1.26 (0.92-1.73) |
| ≥ 10th & < 30th | **1.44 (1.06-1.96)** |
| < 10th | **2.02 (1.44-2.82)** |
| Peak expiratory flow trajectories (n = 866) |  |
| High or upper-moderate | Reference |
| Moderate | **4.80 (2.27-10.16)** |
| Lower-moderate | **5.94 (2.75-12.83)** |
| Low | **8.13 (3.48-19.01)** |

Notes: Boldface: *P* < 0.05; HR: hazard ratio; CI: confidence interval;

For the analyses of peak expiratory flow at baseline, hazard ratios were adjusted for age, sex, race, educational level, marital status, per capita household income, alcohol consumption, body mass index, disability, number of chronic diseases, chronic respiratory disease status, depression, cognitive function, pain, C-reactive protein, and glycosylated haemoglobin A1c.

For the analyses of peak expiratory flow trajectories, there were no participants in the high level trajectory developing frailty during follow-up, therefore, the high level and upper-moderate level trajectory were combined as the reference group. Hazard ratios were adjusted for age, sex, race, educational level, marital status, per capita household income, alcohol consumption, body mass index, disability, number of chronic diseases, depression, cognitive function, pain, total cholesterol, C-reactive protein, and glycosylated haemoglobin A1c.

**Table S8** Diagnostics of models with all possible combinations of polynomial order and number of groups for the analysis of frailty among participants without respiratory diseases at baseline

| **Number of trajectories** | **Polynomial order ^a^** | **BIC (n = 5010) ^b^** | **BIC (n = 1670) ^c^** | **Proportion (%)** | **AvePP ^d^** | **OCC ^e^** | **Entropy** |
| --- | --- | --- | --- | --- | --- | --- | --- |
| 2 | 2 2 | -30005.50 | -30001.11 | 70.18,29.82 | 0.97,0.95 | 14.33,44.30 | 0.881 |
| 3 | 2 2 2 | -29563.98 | -29557.38 | 50.54,35.39,14.07 | 0.94,0.90,0.95 | 16.21,16.33,110.68 | 0.847 |
| 4 | 2 2 2 2 | -29338.50 | -29329.71 | 18.74,45.09,25.27,10.90 | 0.89,0.88,0.91,0.94 | 33.62,9.37,29.47,128.32 | 0.824 |
| 5 | 2 2 2 2 2 | -29220.60 | -29209.62 | 11.14,40.66,25.45,15.62,7.13 | 0.88,0.88,0.86,0.89,0.93 | 59.96,11.21,18.34,45.86,179.08 | 0.823 |
| 5 | 1 1 1 1 1 | -29206.59 | -29198.35 | 11.32,41.08,25.15,15.45,7.01 | 0.88,0.88,0.87,0.90,0.93 | 56.00,10.66,19.30,47.46,185.51 | 0.822 |
| 5 | 1 1 1 1 2 | -29210.78 | -29201.99 | 11.32,41.08,25.15,15.39,7.07 | 0.88,0.88,0.87,0.90,0.93 | 55.97,10.66,19.29,48.39,174.23 | 0.822 |
| **5** | **1 1 1 2 1** | **-29204.12** | **-29195.33** | **11.32,40.42,25.45,15.69,7.13** | **0.88,0.89,0.86,0.89,0.93** | **55.56,11.52,18.26,45.23,180.85** | **0.822** |
| 5 | 1 1 1 2 2 | -29208.37 | -29199.03 | 11.32,40.42,25.45,15.69,7.13 | 0.88,0.89,0.86,0.89,0.93 | 55.54,11.52,18.26,45.20,181.30 | 0.822 |
| 5 | 1 1 2 1 1 | -29210.47 | -29201.68 | 11.32,41.02,25.15,15.57,6.95 | 0.88,0.88,0.87,0.89,0.94 | 56.07,10.75,19.41,45.14,196.22 | 0.822 |
| 5 | 1 1 2 1 2 | -29214.66 | -29205.32 | 11.32,41.02,25.15,15.57,6.95 | 0.88,0.88,0.87,0.89,0.94 | 56.03,10.74,19.40,45.07,197.06 | 0.822 |
| 5 | 1 1 2 2 1 | -29208.21 | -29198.87 | 11.32,40.48,25.45,15.63,7.13 | 0.88,0.89,0.86,0.89,0.93 | 55.54,11.42,18.35,45.92,178.71 | 0.822 |
| 5 | 1 1 2 2 2 | -29212.46 | -29202.57 | 11.32,40.48,25.45,15.63,7.13 | 0.88,0.89,0.86,0.89,0.93 | 55.51,11.42,18.34,45.88,179.17 | 0.822 |
| 5 | 1 2 1 1 1 | -29210.74 | -29201.95 | 11.38,41.08,25.09,15.45,7.01 | 0.88,0.88,0.87,0.90,0.93 | 54.96,10.69,19.49,47.35,184.82 | 0.822 |
| 5 | 1 2 1 1 2 | -29214.94 | -29205.60 | 11.38,41.08,25.09,15.45,7.01 | 0.88,0.88,0.87,0.90,0.93 | 54.93,10.69,19.48,47.26,185.54 | 0.822 |
| 5 | 1 2 1 2 1 | -29208.28 | -29198.94 | 11.38,40.54,25.27,15.69,7.13 | 0.87,0.89,0.86,0.89,0.93 | 54.52,11.36,18.81,45.12,180.24 | 0.822 |
| 5 | 1 2 1 2 2 | -29212.53 | -29202.64 | 11.32,40.60,25.27,15.69,7.13 | 0.88,0.89,0.86,0.89,0.93 | 55.84,11.27,18.81,45.09,180.70 | 0.822 |
| 5 | 1 2 2 1 1 | -29214.66 | -29205.32 | 11.38,41.02,25.09,15.57,6.95 | 0.88,0.88,0.87,0.89,0.94 | 54.96,10.77,19.60,45.07,195.66 | 0.822 |
| 5 | 1 2 2 1 2 | -29218.85 | -29208.97 | 11.38,41.02,25.09,15.57,6.95 | 0.88,0.88,0.87,0.89,0.94 | 54.93,10.77,19.59,45.00,196.49 | 0.822 |
| 5 | 1 2 2 2 1 | -29212.39 | -29202.51 | 11.38,40.48,25.39,15.63,7.13 | 0.87,0.89,0.86,0.89,0.93 | 54.46,11.45,18.51,45.83,178.30 | 0.822 |
| 5 | 1 2 2 2 2 | -29216.64 | -29206.21 | 11.32,40.54,25.39,15.63,7.13 | 0.88,0.89,0.86,0.89,0.93 | 55.77,11.35,18.50,45.80,178.76 | 0.822 |
| 5 | 2 1 1 1 1 | -29210.58 | -29201.79 | 11.32,40.96,25.27,15.45,7.01 | 0.88,0.88,0.86,0.90,0.93 | 55.73,10.86,18.93,47.52,185.96 | 0.822 |
| 5 | 2 1 1 1 2 | -29214.77 | -29205.43 | 11.32,40.96,25.27,15.39,7.07 | 0.88,0.88,0.86,0.90,0.93 | 55.70,10.85,18.92,48.46,174.64 | 0.822 |
| 5 | 2 1 1 2 1 | -29208.11 | -29198.77 | 11.32,40.42,25.45,15.69,7.13 | 0.88,0.89,0.86,0.89,0.93 | 55.29,11.55,18.30,45.30,181.24 | 0.822 |
| 5 | 2 1 1 2 2 | -29212.36 | -29202.47 | 11.32,40.42,25.45,15.69,7.13 | 0.88,0.89,0.86,0.89,0.93 | 55.27,11.55,18.29,45.27,181.69 | 0.822 |
| 5 | 2 1 2 1 1 | -29214.46 | -29205.12 | 11.32,41.02,25.15,15.57,6.95 | 0.88,0.88,0.87,0.89,0.94 | 55.79,10.77,19.44,45.21,196.70 | 0.822 |
| 5 | 2 1 2 1 2 | -29218.65 | -29208.77 | 11.32,41.02,25.15,15.57,6.95 | 0.88,0.88,0.87,0.89,0.94 | 55.76,10.77,19.43,45.13,197.55 | 0.822 |
| 5 | 2 1 2 2 1 | -29212.20 | -29202.31 | 11.32,40.42,25.51,15.63,7.13 | 0.88,0.89,0.86,0.89,0.93 | 55.27,11.55,18.19,45.98,179.11 | 0.822 |
| 5 | 2 1 2 2 2 | -29216.45 | -29206.01 | 11.32,40.42,25.51,15.63,7.13 | 0.88,0.89,0.86,0.89,0.93 | 55.24,11.54,18.18,45.95,179.54 | 0.822 |
| 5 | 2 2 1 1 1 | -29214.70 | -29205.36 | 11.32,41.14,25.09,15.45,7.01 | 0.88,0.88,0.87,0.90,0.93 | 56.13,10.64,19.52,47.40,185.16 | 0.822 |
| 5 | 2 2 1 1 2 | -29218.90 | -29209.01 | 11.32,41.14,25.09,15.39,7.07 | 0.88,0.88,0.87,0.90,0.93 | 56.10,10.64,19.51,48.34,173.92 | 0.823 |
| 5 | 2 2 1 2 1 | -29212.23 | -29202.35 | 11.20,40.60,25.39,15.69,7.13 | 0.88,0.89,0.86,0.89,0.93 | 58.55,11.31,18.45,45.17,180.57 | 0.822 |
| 5 | 2 2 1 2 2 | -29216.48 | -29206.04 | 11.14,40.66,25.39,15.69,7.13 | 0.88,0.88,0.86,0.89,0.93 | 60.04,11.21,18.45,45.14,181.03 | 0.822 |
| 5 | 2 2 2 1 1 | -29218.62 | -29208.73 | 11.32,41.02,25.15,15.57,6.95 | 0.88,0.88,0.87,0.89,0.94 | 56.12,10.80,19.42,45.12,196.09 | 0.822 |
| 5 | 2 2 2 1 2 | -29222.82 | -29212.38 | 11.32,41.02,25.15,15.57,6.95 | 0.88,0.88,0.87,0.89,0.94 | 56.09,10.80,19.41,45.05,196.91 | 0.823 |
| 5 | 2 2 2 2 1 | -29216.35 | -29205.92 | 11.20,40.60,25.45,15.63,7.13 | 0.88,0.89,0.86,0.89,0.93 | 58.46,11.30,18.35,45.89,178.64 | 0.823 |

Notes: When the polynomial order is three, the results were not statistically significant (P > 0.05). Although the linear term was not statistically significant, the linear term was retained according to previous studies.

^a^ Trajectory shapes; 1 = linear; 2 = quadratic; 3 = cubic; ^b^ BIC = Bayesian information criterion (for the total number of observations); ^c^ BIC = Bayesian information criterion (for the total number of participants); ^d^ Average group posterior probability. An AvePP greater than 0.7 for all groups is recommended; ^e^ Odds of correct classification. An OCC of 5 or more is recommended for all groups.

**Table S9** Association of peak expiratory flow at baseline and its trajectories with frailty risk among participants without respiratory diseases at baseline

| Peak expiratory flow | HR (95%CI) |
| --- | --- |
| Measured values at baseline (n = 5181) |  |
| Q_1_ | **2.96 (2.25-3.88)** |
| Q_2_ | **2.27 (1.72-2.98)** |
| Q_3_ | **2.01 (1.55-2.61)** |
| Q_4_ | **1.29 (1.01-1.67)** |
| Q_5_ | Reference |
| Standard residual percentiles at baseline (n = 5181) |  |
| ≥ 80th | Reference |
| ≥ 50th & < 80th | **1.24 (1.01-1.53)** |
| ≥ 30th & < 50th | **1.40 (1.12-1.75)** |
| ≥ 10th & < 30th | **1.52 (1.22-1.88)** |
| < 10th | **2.15 (1.69-2.73)** |
| Peak expiratory flow trajectories (n = 1670) |  |
| High | Reference |
| Upper-moderate | 2.38 (0.92-6.14) |
| Moderate | **3.19 (1.28-7.95)** |
| Lower-moderate | **4.02 (1.63-9.92)** |
| Low | **4.93 (1.93-12.64)** |

Notes: Boldface: *P* < 0.05; HR: hazard ratio; CI: confidence interval;

For the analyses of peak expiratory flow at baseline, hazard ratios were adjusted for age, sex, race, educational level, marital status, per capita household income, alcohol consumption, body mass index, disability, number of chronic diseases, depression, cognitive function, pain, total cholesterol, C-reactive protein, and glycosylated haemoglobin A1c.

For the analyses of peak expiratory flow trajectories, hazard ratios were adjusted for age, race, educational level, marital status, per capita household income, alcohol consumption, body mass index, disability, number of chronic diseases, depression, cognitive function, pain, total cholesterol, C-reactive protein, and glycosylated haemoglobin A1c.

**Table S10** Diagnostics of models with all possible combinations of polynomial order and number of groups for the analysis of frailty among participants without missing data of Fried frailty phenotype

| **Number of trajectories** | **Polynomial order ^a^** | **BIC (n = 4506) ^b^** | **BIC (n = 1502) ^c^** | **Proportion (%)** | **AvePP ^d^** | **OCC ^e^** | **Entropy** |
| --- | --- | --- | --- | --- | --- | --- | --- |
| 2 | 2 2 | -27048.22 | -27043.83 | 69.51,30.49 | 0.97,0.95 | 13.88,41.31 | 0.875 |
| 3 | 2 2 2 | -26642.86 | -26636.27 | 50.80,36.48,12.72 | 0.94,0.90,0.95 | 16.05,16.23,119.44 | 0.850 |
| 4 | 2 2 2 2 | -26401.61 | -26392.82 | 18.64,45.27,25.90,10.19 | 0.89,0.90,0.92,0.95 | 36.15,10.62,31.92,154.96 | 0.839 |
| 5 | 2 2 2 2 2 | -26303.32 | -29292.33 | 13.12,40.74,24.17,15.51,6.46 | 0.90,0.88,0.86,0.89,0.93 | 57.92,10.86,19.70,45.73,204.13 | 0.823 |
| 5 | 1 1 1 1 1 | -26291.58 | -26283.34 | 13.32,40.75,24.30,15.31,6.32 | 0.89,0.88,0.86,0.89,0.93 | 54.54,11.11,19.48,46.10,198.01 | 0.823 |
| 5 | 1 1 1 1 2 | -26295.49 | -26286.70 | 13.32,40.75,24.30,15.25,6.39 | 0.89,0.88,0.86,0.89,0.93 | 54.53,11.10,19.47,47.10,183.33 | 0.823 |
| **5** | **1 1 1 2 1** | **-26287.26** | **-26278.47** | **13.18,40.68,24.03,15.65,6.46** | **0.89,0.88,0.86,0.89,0.94** | **55.94,10.86,20.07,44.59,209.03** | **0.823** |
| 5 | 1 1 1 2 2 | -26291.34 | -26282.00 | 13.18,40.68,24.03,15.65,6.46 | 0.89,0.88,0.86,0.89,0.94 | 55.84,10.84,20.04,44.57,210.77 | 0.823 |
| 5 | 1 1 2 1 1 | -26295.22 | -26286.43 | 13.45,40.68,24.23,15.38,6.26 | 0.89,0.89,0.86,0.89,0.93 | 52.27,11.28,19.94,43.88,206.07 | 0.823 |
| 5 | 1 1 2 1 2 | -26299.13 | -26289.80 | 13.38,40.75,24.23,15.31,6.32 | 0.89,0.88,0.86,0.89,0.93 | 53.63,11.18,19.93,44.78,189.80 | 0.823 |
| 5 | 1 1 2 2 1 | -26291.25 | -26281.91 | 13.18,40.68,24.17,15.51,6.46 | 0.89,0.88,0.86,0.89,0.93 | 56.13,10.88,19.75,45.94,203.27 | 0.823 |
| 5 | 1 1 2 2 2 | -26295.32 | -26285.43 | 13.18,40.68,24.10,15.58,6.46 | 0.89,0.88,0.86,0.89,0.93 | 56.02,10.87,19.96,44.89,204.86 | 0.823 |
| 5 | 1 2 1 1 1 | -26295.38 | -26286.59 | 13.45,40.61,24.30,15.31,6.32 | 0.89,0.89,0.86,0.89,0.93 | 52.26,11.40,19.46,45.78,195.97 | 0.823 |
| 5 | 1 2 1 1 2 | -26299.29 | -26289.95 | 13.45,40.61,24.30,15.25,6.39 | 0.89,0.89,0.86,0.89,0.93 | 52.25,11.39,19.46,46.76,181.38 | 0.823 |
| 5 | 1 2 1 2 1 | -26291.06 | -26281.73 | 13.18,40.68,24.03,15.65,6.46 | 0.90,0.88,0.86,0.89,0.93 | 56.44,10.94,20.04,44.29,207.19 | 0.823 |
| 5 | 1 2 1 2 2 | -26295.14 | -26285.25 | 13.18,40.68,24.03,15.65,6.46 | 0.90,0.88,0.86,0.89,0.94 | 56.34,10.92,20.01,44.27,208.88 | 0.823 |
| 5 | 1 2 2 1 1 | -26299.09 | -26289.75 | 13.52,40.61,24.23,15.38,6.26 | 0.89,0.89,0.86,0.89,0.93 | 51.34,11.47,19.92,43.65,204.40 | 0.823 |
| 5 | 1 2 2 1 2 | -26303.00 | -26293.12 | 13.52,40.61,24.23,15.31,6.32 | 0.89,0.89,0.86,0.89,0.93 | 51.33,11.47,19.92,44.53,188.26 | 0.823 |
| 5 | 1 2 2 2 1 | -26295.10 | -26285.21 | 13.18,40.68,24.17,15.51,6.46 | 0.90,0.88,0.86,0.89,0.93 | 56.56,10.97,19.72,45.70,202.04 | 0.823 |
| 5 | 1 2 2 2 2 | -26299.17 | -26288.73 | 13.18,40.68,24.17,15.51,6.46 | 0.90,0.88,0.86,0.89,0.93 | 56.46,10.95,19.70,45.67,203.61 | 0.823 |
| 5 | 2 1 1 1 1 | -26295.75 | -26286.96 | 13.32,40.75,24.30,15.31,6.32 | 0.89,0.88,0.86,0.89,0.93 | 54.41,11.12,19.49,46.15,198.36 | 0.823 |
| 5 | 2 1 1 1 2 | -26299.66 | -26290.32 | 13.32,40.75,24.30,15.25,6.39 | 0.89,0.88,0.86,0.89,0.93 | 54.40,11.11,19.48,47.16,183.67 | 0.823 |
| 5 | 2 1 1 2 1 | -26291.43 | -26282.09 | 13.12,40.75,24.03,15.65,6.46 | 0.90,0.88,0.86,0.89,0.94 | 57.32,10.77,20.08,44.64,209.34 | 0.823 |
| 5 | 2 1 1 2 2 | -26295.50 | -26285.62 | 13.12,40.75,24.03,15.65,6.46 | 0.90,0.88,0.86,0.89,0.94 | 57.22,10.75,20.05,44.61,211.11 | 0.823 |
| 5 | 2 1 2 1 1 | -26299.39 | -26290.05 | 13.45,40.68,24.23,15.38,6.26 | 0.89,0.89,0.86,0.89,0.93 | 52.15,11.29,19.95,43.93,206.42 | 0.823 |
| 5 | 2 1 2 1 2 | -26303.30 | -26293.42 | 13.45,40.68,24.23,15.31,6.32 | 0.89,0.89,0.86,0.89,0.93 | 52.16,11.28,19.94,44.84,190.23 | 0.823 |
| 5 | 2 1 2 2 1 | -26295.41 | -26285.53 | 13.18,40.68,24.17,15.51,6.46 | 0.89,0.88,0.86,0.89,0.93 | 55.97,10.89,19.77,45.99,203.55 | 0.823 |
| 5 | 2 1 2 2 2 | -26299.49 | -26289.05 | 13.12,40.75,24.10,15.58,6.46 | 0.90,0.88,0.86,0.89,0.93 | 57.40,10.78,19.98,44.93,205.14 | 0.823 |
| 5 | 2 2 1 1 1 | -26299.52 | -26290.18 | 13.45,40.61,24.30,15.31,6.32 | 0.89,0.89,0.86,0.89,0.93 | 52.12,11.41,19.48,45.83,196.31 | 0.823 |
| 5 | 2 2 1 1 2 | -26303.44 | -26293.55 | 13.45,40.61,24.30,15.25,6.39 | 0.89,0.89,0.86,0.89,0.93 | 52.13,11.40,19.46,46.82,181.85 | 0.823 |
| 5 | 2 2 1 2 1 | -26295.21 | -26285.32 | 13.12,40.75,24.03,15.65,6.46 | 0.90,0.88,0.86,0.89,0.93 | 57.87,10.86,20.06,44.34,207.51 | 0.823 |
| 5 | 2 2 1 2 2 | -26299.29 | -26288.85 | 13.12,40.75,24.03,15.65,6.46 | 0.90,0.88,0.86,0.89,0.94 | 57.78,10.84,20.03,44.32,209.29 | 0.823 |
| 5 | 2 2 2 1 1 | -26303.24 | -26293.36 | 13.52,40.61,24.23,15.38,6.26 | 0.89,0.89,0.86,0.89,0.93 | 51.21,11.48,19.94,43.70,204.76 | 0.823 |
| 5 | 2 2 2 1 2 | -26307.16 | -26296.72 | 13.52,40.61,24.23,15.31,6.32 | 0.89,0.89,0.86,0.89,0.93 | 51.20,11.48,19.94,44.59,188.59 | 0.823 |
| 5 | 2 2 2 2 1 | -26299.24 | -26288.81 | 13.12,40.75,24.17,15.51,6.46 | 0.90,0.88,0.86,0.89,0.93 | 58.00,10.88,19.74,45.75,202.35 | 0.823 |

Notes: When the polynomial order is three, the results were not statistically significant (P > 0.05). Although the linear term was not statistically significant, the linear term was retained according to previous studies.

^a^ Trajectory shapes; 1 = linear; 2 = quadratic; 3 = cubic; ^b^ BIC = Bayesian information criterion (for the total number of observations); ^c^ BIC = Bayesian information criterion (for the total number of participants); ^d^ Average group posterior probability. An AvePP greater than 0.7 for all groups is recommended; ^e^ Odds of correct classification. An OCC of 5 or more is recommended for all groups.

**Table S11** Association of peak expiratory flow at baseline and its trajectories with frailty risk among participants without missing values of Fried frailty phenotype

| Peak expiratory flow | HR (95%CI) |
| --- | --- |
| Measured values at baseline (n = 4431) |  |
| Q_1_ | **2.68 (1.99-3.61)** |
| Q_2_ | **2.18 (1.63-2.91)** |
| Q_3_ | **1.57 (1.17-2.11)** |
| Q_4_ | 1.26 (0.96-1.65) |
| Q_5_ | Reference |
| Standard residual percentiles at baseline (n = 4431) |  |
| ≥ 80th | Reference |
| ≥ 50th & < 80th | 1.24 (0.98-1.56) |
| ≥ 30th & < 50th | **1.37 (1.07-1.75)** |
| ≥ 10th & < 30th | **1.50 (1.18-1.91)** |
| < 10th | **2.06 (1.56-2.72)** |
| Peak expiratory flow trajectories (n = 1502) |  |
| High | Reference |
| Upper-moderate | 1.97 (0.75-5.18) |
| Moderate | **3.08 (1.21-7.83)** |
| Lower-moderate | **4.62 (1.79-11.93)** |
| Low | **6.79 (2.51-18.37)** |

Notes: Boldface: *P* < 0.05; HR: hazard ratio; CI: confidence interval;

For the analyses of peak expiratory flow at baseline, hazard ratios were adjusted for age, sex, race, educational level, marital status, per capita household income, smoking status, alcohol consumption, body mass index, disability, number of chronic diseases, chronic respiratory disease status, depression, cognitive function, pain, high-density lipoprotein cholesterol, total cholesterol, C-reactive protein, and glycosylated haemoglobin A1c.

For the analyses of peak expiratory flow trajectories, hazard ratios were adjusted for age, sex, race, educational level, marital status, per capita household income, alcohol consumption, body mass index, disability, number of chronic diseases, chronic respiratory disease status, depression, cognitive function, pain, high-density lipoprotein cholesterol, total cholesterol, C-reactive protein, and glycosylated haemoglobin A1c.

**References:**

1.Wu C K, Geldhof G J, Xue Q L, Kim D H, Newman A B, Odden M C. Development, construct validity, and predictive validity of a continuous frailty scale: results from 2 large us cohorts. Am J Epidemiol. 2018;187:1752-1762.

2.Fragoso C, Gahbauer E A, Van Ness P H, Gill T M. Reporting peak expiratory flow in older persons. J Gerontol a-Biol. 2007;62:1147-1151.

3.Donahue P T, Xue Q L, Carlson M C. Peak expiratory flow predicts incident dementia in a representative sample of us older adults: the national health and aging trends study (nhats). J Gerontol a-Biol. 2023;78:1427-1435.

4.Rodin R, Smith A K, Espejo E, Gan S Q, Boscardin W J, Hunt L J, et al. Mortality and function after widowhood among older adults with dementia, cancer, or organ failure. Jama Netw Open. 2024;7.

5.Zivin K, Llewellyn D J, Lang I A, Vijan S, Kabeto M U, Miller E M, et al. Depression among older adults in the united states and england. Am J Geriat Psychiat. 2010;18:1036-1044.

6.Martinez M, Dawson A Z, Lu K, Walker R J, Egede L E. Effect of cognitive impairment on risk of death in hispanic/latino adults over the age of 50 residing in the united states with and without diabetes: data from the health and retirement study 1995-2014. Alzheimers Dement. 2022;18:1616-1624.
